# Glucosinolate Metabolites and Brain Health: An Updated Review on Their Potential Benefits in Neurodegenerative, Neurodevelopmental, and Psychiatric Disorders

**DOI:** 10.3390/antiox14070818

**Published:** 2025-07-02

**Authors:** Claudia Muscarà, Agnese Gugliandolo, Emanuela Mazzon, Gabriella Calì

**Affiliations:** 1 IRCCS Centro Neurolesi “Bonino-Pulejo”, Via Provinciale Palermo, Contrada Casazza, 98124 Messina, Italy; claudia.muscara@irccsme.it (C.M.); gabriella.cali@irccsme.it (G.C.); 2 Department of Medical, Oral and Biotechnological Sciences, University “G. D’Annunzio” Chieti-Pescara, 66100 Chieti, Italy

**Keywords:** glucosinolate, isothiocyanate, Alzheimer’s disease, Parkinson’s disease, multiple sclerosis, autism spectrum disorder, schizophrenia, depression, anxiety, epilepsy, antioxidant effects

## Abstract

Neurodegenerative, neurodevelopmental, and psychiatric disorders, as well as epilepsy, affect millions of people. Due to their impact on patients’ quality of life, they represent a major health issue. Natural compounds are arising as new treatments for these diseases. Particularly, glucosinolates (GLS) are secondary metabolites found in Cruciferae family plants. Their basic structure consists of a glucose unit linked to a thiohydroximate-O-sulfonate group and an aliphatic, aralkyl, or indolyl side chain, depending on their precursor amino acid. Specifically, aliphatic GLS derive from methionine, aromatic ones from phenylalanine, and indolic ones from tryptophan. Myrosinase (thioglucoside glucohydrolase) is the crucial enzyme for GLS degradation, leading to the production of isothiocyanates (ITCs). ITCs attracted considerable scientific interest for their protective effects against various diseases, thanks to their antioxidant, anti-inflammatory, and neuroprotective properties. Here, we collected the latest evidence regarding ITC effects in neurodegenerative, neurodevelopmental, and psychiatric disorders, including preclinical and clinical studies published in the last decade. These studies evidenced ITCs’ neuroprotective effects, exerted mainly through antioxidant and anti-inflammatory mechanisms. Thus, ITCs’ integration, also through the diet, may represent a safe and efficacious strategy to improve health and limit the risk of neurological and psychiatric disorders. However, new large-scale trials are needed to determine their therapeutic potential, particularly for diseases with no clinical evidence.

## 1. Introduction

Neurodegenerative diseases, neurodevelopmental disorders, psychiatric disorders, and epilepsy represent a major global health issue, affecting millions of people and significantly impacting patients’ quality of life, while also generating substantial social and economic costs for families and healthcare systems. Neurodegenerative diseases, including Alzheimer’s disease (AD), Parkinson’s disease (PD), multiple sclerosis (MS), and other neurodegenerative conditions, are characterized by the progressive loss of neurons in specific brain areas. This irreversible process leads to brain damage, resulting in severe disabilities and premature death [1]. Similarly, neurodevelopmental disorders, such as autism spectrum disorder (ASD) and schizophrenia, affect brain development and severely impair individuals’ ability to socially interact, communicate, and develop cognitive skills. Psychiatric disorders, such as depression and anxiety disorders, are among the most prevalent mental health conditions and constitute one of the leading causes of disability worldwide [2]. Mental disorders diagnosis and classification are based on the Diagnostic and Statistical Manual of Mental Disorders (DSM-5) criteria, which includes a description of symptoms and their duration [3]. Neurodegenerative diseases, neurodevelopmental disorders, and psychiatric disorders share some common features. Indeed, inflammation and oxidative stress play a key role in the pathogenesis of all these diseases [1,4]. Moreover, given the progressive aging of the global population, the increased life expectancy, and the growing incidence of these disorders, the number of individuals affected is expected to rise significantly, highlighting the urgent need to find effective therapeutic solutions [5]. In this context, the search for new therapeutic agents is increasingly crucial, as current treatments are not always able to effectively address the underlying biological mechanisms of these diseases.

Given the main role played by oxidative stress and inflammation, molecules capable of counteracting these mechanisms could be particularly promising in treating such pathological conditions. Among natural compounds, glucosinolate (GLS) derivatives, namely isothiocyanates (ITCs), are emerging as potential beneficial agents to counteract neurodegenerative diseases, neurodevelopmental disorders, psychiatric disorders, and epilepsy. GLS are secondary metabolites found abundantly in plants of the Cruciferae family (Brassicaceae), including broccoli, cauliflower, cabbage, and other cruciferous vegetables. GLS derivatives, obtained through hydrolysis, have attracted considerable scientific interest for their potential protective effects against a wide range of diseases, thanks to their significant antioxidant, anti-inflammatory, and neuroprotective properties [6].

In particular, the ITC ability to activate the nuclear factor 2-related, erythroid-derived factor (Nrf2)/via antioxidant response element (ARE) and to inhibit the nuclear factor (NF)-κB activation [7] may contribute to their antioxidant and anti-inflammatory functions and, consequently, to their neuroprotective effect.

The ITC sulforaphane (SFN) demonstrated powerful neuroprotective effects by modulating oxidative stress and inflammation, as well as promoting mitochondrial function and synaptic protection [8]. Moreover, SFN exhibited neuroprotective effects against oxidative stress by inhibiting the activity of NADPH oxidase Nox4 in endothelial cells and astrocytes [9].

Thus, the benefits of these compounds suggest that they may represent a new strategy to treating neurodegenerative diseases, neurodevelopmental disorders, psychiatric disorders, and epilepsy, offering complementary or alternative therapies to traditional pharmacological options. In vitro and animal model studies play a crucial role in understanding the mechanisms underlying the beneficial effects of GLS derivatives, providing the basis for future clinical studies in humans. However, the number of randomized clinical trials in this field is still limited, highlighting the importance of leveraging preclinical evidence to guide clinical research. Therefore, this review aims to provide an in-depth update on the latest evidence regarding the effects of GLS derivatives in neurodegenerative diseases, neurodevelopmental disorders, and psychiatric disorders, including all preclinical and clinical studies published between 2014 and 2024.

## 2. Origin, Structure, and Biosynthesis of Glucosinolates and Their Derivatives

**Figure 1:**
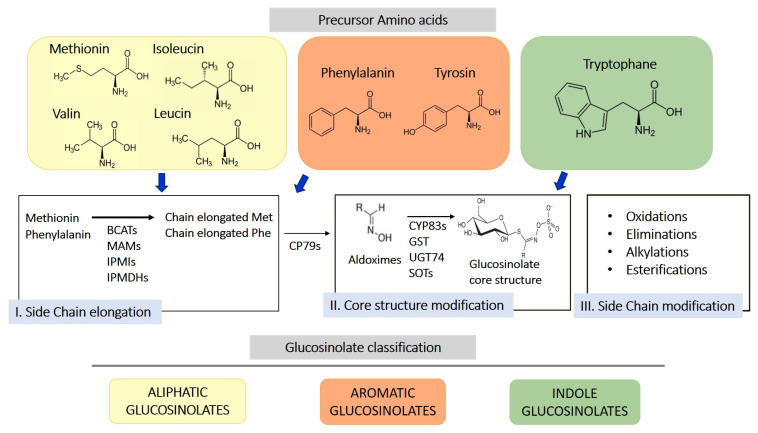
Schematic representation of biosynthesis of glucosinolates from 3 different amino acid precursors. Depending on the amino acid precursor, glucosinolate can be divided in three different chemical classes: aliphatic, aromatic, and indole glucosinolates. The biosynthesis process is divided into three distinct steps, side chain elongation (for methionine and phenylalanine-derived glucosinolates), core structure formation, and secondary modification. BCAT, branched-chain amino acid aminotransferase; MAM, methylthioalkylmalate synthase; IPMI, isopropylmalate isomerase; IPMDHs, isopropylmalate dehydrogenases; CP79s, cytochromes 79; GST, glutathione-S-transferase; UGT74, glucosyltransferase 74; SOTs, sulfotransferases.

GLS are sulfur-containing secondary metabolites primarily found in plants of the Brassicaceae family, such as broccoli, mustard, cabbage, and cauliflower [10]. Their basic structure consists of a glucose unit linked to a thiohydroximate-O-sulfonate group and an aliphatic, aralkyl, or indolyl side chain (R) [11]. The side chain varies depending on the originating amino acid, classifying GLS into three main groups: aliphatic, aromatic, and indolic. Specifically, aliphatic GLS derive from methionine, aromatic ones from phenylalanine, and indolic ones from tryptophan. The structure and variability of these side chains are key determinants of the chemical and biological properties of the GLS derivates produced through their hydrolysis. More than 200 side groups have been identified [11]. The biosynthesis of GLS is a complex process involving multiple stages (Figure 1). GLS originate from precursor amino acids such as methionine, phenylalanine, tryptophan, and others, undergoing a series of enzymatic reactions, including N-hydroxylation, oxidative decarboxylation, and oxidation, leading to the formation of a sulfur-containing structure [10]. This biosynthetic process can be regulated by environmental factors and genetic variables, determining the concentration of GLS in plants. One of the key genes involved in GLS synthesis is CYP79, which determines which amino acid is used for GLS formation. However, other enzymes, including CYP83s, GSTF, GGP1, SUR1, UGT, SOTs, participate in their formation. The genetic architecture is complex, and the creation of new genes/enzymes plays a role in expanding GLS’s chemical diversity. Thanks to the study of new genomes and to the mechanistic assessment of GLS diversity in the Brassicales, how chemical novelty may arise is beginning to become clearer. Interestingly, small-scale duplications like tandem or distal events play a role in the formation of metabolic novelty. Also, gene loss is important in metabolic diversity across the entire genera [12].

It is important to note that studies demonstrated a wide variability in GLS profile in both cultivated Brassica species and crop wild relatives (CWRs) [13,14] in regards to GLS types and content, with differences in total GLS concentrations of approximately 10-fold between different accessions.

These compounds play a defensive role in plants, protecting them from environmental stress, pathogens, and parasites [15]. GLSs are stable molecules within plant cells. However, when plant tissue is damaged (e.g., through chewing or crushing), GLSs come into contact with the myrosinase (MYR) enzyme, which catalyzes their hydrolysis. This enzyme is typically stored separately from GLS in plant species [16]. The hydrolysis process leads to the formation of a β-D-glucose molecule and an unstable aglycone (thiohydroximate-O-sulfonate). Spontaneous rearrangements of these intermediates generate sulfate ions and metabolites with structures that vary depending on the GLS side chain and the physicochemical conditions. Under acidic pH and a Fe^2+^-rich environment, nitrile formation is favored [17]. At neutral pH, ITC formation is preferred; these compounds are unstable and easily break down into thiocyanate ion and indole-3-carbinol [18]. If the side chain has a β-hydroxy function, ITCs spontaneously cyclize into oxazolidine-2-thione [10,19,20]. Although GLS are biochemically inactive, their hydrolysis by MYR transforms them into biologically active compounds, such as ITCs, which are known for their multiple health benefits. ITCs, deriving from GLS hydrolysis, are a class of molecules characterized by the R-N=C=S general formula, where R represents an alkyl or aryl group. Structural variations in GLS influence the formation of different types of ITCs through enzymatic hydrolysis. Specifically, glucoraphanin (GRA) gives rise to SFN, sinigrin (SIN) generates allyl isothiocyanate (AITC), gluconasturtiin (GST) produces phenethyl isothiocyanate (PEITC), glucoerucin (GER) converts into erucin (ER), glucotropaeolin (GTL) leads to benzyl isothiocyanate (BITC), and glucomoringin (GMG) is converted into moringin (MOR) [21]. The functional group responsible for the biological activity of ITCs is the central electrophilic carbon of the R-N=C=S group, often referred to as the reactive site, which is particularly susceptible to nucleophilic attack [22]. Many studies have highlighted the antioxidant potential of ITCs, particularly their ability to stimulate phase II enzymes, although research demonstrating their direct antioxidant activity remains limited [23]. Additionally, ITCs exhibit other beneficial properties, including anti-inflammatory, antimicrobial, neuroprotective, and cardioprotective activities [24]. These compounds are generally considered safe, with no significant adverse effects observed in humans [25,26].

## 3. Metabolism and Bioavailability of Isothiocyanates

In humans, ITC metabolism begins with their conjugation to glutathione (GSH) through the action of glutathione S-transferases (GSTs) [27]. Subsequently, the enzyme gamma-glutamyl transpeptidase (GTP) catalyzes the isothiocyanate-Cys-Gly production, which is then converted into Cys-isothiocyanate by the cysteinylglycinase (CG) enzyme. Finally, N-acetyl transferase (NAT) facilitates the N-acetylcysteine isothiocyanate (NAC-ITC) formation [28,29]. ITCs’ bioavailability is influenced by several factors, including food matrix composition, the type of source material (food or supplement), preparation method (raw or cooked), storage, and intestinal metabolism [8]. After ingestion, some studies showed that intact GLS can be partially absorbed in the stomach, while those not absorbed continue through the gastrointestinal tract to the small intestine, where they may be hydrolyzed by plant-derived MYR, and the resulting products are absorbed [8]. When MYR is inactivated, unmetabolized GLS reach the colon, where they are processed by the gut microbiota due to their hydrophilic nature (thioglucosidic and sulfate groups). Heat treatment of plant matrices tends to denature MYR, with an intensity depending on temperature and cooking time, regardless of the method used, such as boiling [30], steaming [31,32], or microwaving [33]. Despite the thermal destruction of MYR enzymatic activity, consuming cooked vegetables still results in the intake of GLS hydrolysis derivatives. Data showed that raw cruciferous consumption improved ITCs’ bioavailability. Boiling significantly reduced GLS levels, while steam cooking, microwaving, and stir-frying did not cause significant changes in their content. Storage reduced GLS content compared to fresh harvest broccoli [8]. Clinical studies showed higher bioavailability of ITCs compared to GLS, with significantly increased urinary excretion in subjects consuming ITCs directly versus those ingesting GLS, reflecting the necessity of enzymatic conversion before absorption [34,35]. Moreover, the presence of MYR in vegetable preparations directly affects ITCs’ bioavailability, with fresh broccoli producing three times higher levels than cooked broccoli [31]. MYR activity is therefore crucial for ITC absorption, as demonstrated by comparisons between fresh broccoli sprouts and GLS-rich broccoli powders lacking MYR, which showed lower conversion and delayed urinary excretion of ITCs [36]. These findings highlight the importance of preparation methods and dietary composition in the pharmacology of ITCs, emphasizing the need for further research to optimize their bioavailability and clinical applications [37].

## 4. Neuroinflammation and Oxidative Stress

Neuroinflammation is a complex mechanism that occurs in the central nervous system (CNS) as a result of brain damage that leads to the activation of resident cells, including astrocytes, impairing their neuroprotective role and leading to the release of inflammatory mediators [38]. Oxidative stress is a consequence of the unregulated release of reactive oxygen species (ROS) that, in turn, triggers oxidative deterioration of molecules leading to several disorders. Neuroinflammation and oxidative stress are major players in the onset and progression of neurodegenerative disorders and are deeply linked. Indeed, inflammatory cells produce ROS increasing oxidative stress that, in turn, increase pro-inflammatory mediators [1].

In recent years, researchers focused on the use of natural compounds that modulate these processes. In particular, ITCs, due to their ability to activate the Nrf2 pathway, may act as both antioxidants and modulators of inflammation [7]. In addition, ITCs reduce inflammation and apoptosis through several mechanisms [7]. These molecules can reduce neuroinflammation inhibiting NF-κB translocation, pro-inflammatory cytokines production, as well as oxidative species generation and apoptotic neuronal death [7].

### Preclinical Studies on Glucosinolate or Isothiocyanates in Neuroinflammation and Oxidative Stress

The use of ITCs could be an important approach to contrast neuroinflammation through the modulation of different pathways. An in vitro study of lipopolysaccharide (LPS)-induced inflammation showed that SFN boosted the release of an-ti-inflammatory cytokines (interleukin (IL)-10, IL-4) and inhibited inflammatory me-diators, such as inducible nitric oxide synthase (iNOS), cyclooxygenase (COX)-2, nitric oxide (NO), prostaglandin E2 (PGE2), and pro-inflammatory cytokines by attenuating the pJNK/JNK pathway [39]. Similarly, Qin and colleagues observed that SFN inhibited necroptosis by blocking the release of inflammatory mediators and preventing NF-κB activation in LPS-induced BV-2 microglia cells [40]. SFN exerted a protective action in LPS- and ATP-induced N9 microglial cells by inhibiting inflammasome activation and pyroptotic death [41]. SFN exerted a neuroprotective effect in a model of methylglyox-al (MGO)-induced neuroinflammation, a toxic byproduct of glucose degradation, in-hibiting pro-inflammatory cytokine production, blocking NF-κB translocation, and de-creasing ROS [42]. Additionally, SFN had a neuroprotective effect on both adult and aged mouse microglial cells, reducing the release of pro-inflammatory cytokines by ac-tivating Nrf2. These findings suggested that SFN was able to control inflammation and may help to reduce age-related cognitive decline [43]. SFN was also able to prevent the mitochondrial dysfunctions promoted by chlorpyrifos (CPF), an inhibitor of acetylcho-linesterase, or LPS in SH-SY5Y and microglia BV-2 cells, respectively, increasing heme oxygenase-1 (HO-1) enzyme [44]. Moreover, SFN stimulates the elongation of micro-glia cells, both in vitro and in vivo, counteracting LPS-induced abnormalities and promoting a shift from an amoeboid (pro-inflammatory) to a more branched (an-ti-inflammatory) form, reprogramming the microglia towards a less inflammatory state [45]. In another study, Wang and colleagues demonstrated that SFN reduced neuroinflammation both in vitro and in vivo. In vitro, using an LPS-induced BV-2 cell model, SFN inhibited the NF-κB pathway by upregulating Cezanne, a deubiquitinating enzyme. In vivo, SFN improved neurocognitive function by modulating the ubiquiti-nation of TRAF6 and RIP1 through Cezanne, leading to the inhibition of the NF-κB pathway [46]. Accordingly, the treatment with SFN improved learning and memory deficits in an LPS in vivo model through the modulation of the mTOR pathway, as well as increasing the expression of brain-derived neurotrophic factor (BDNF), a protein involved in the process of synaptic formation [47]. The neuroprotective effects of SFN were demonstrated in an in vitro and in vivo study using an Okadaic Acid (OKA)-induced model. OKA is a potent selective inhibitor of serine/threonine phos-phatase 1 (PP1) and 2 (PP2A), and in vivo studies showed that the administration of OKA causes mitochondrial dysfunction, oxidative stress, and cell death, in addition to neuroinflammatory processes that compromise memory. SFN reduced levels of pro-inflammatory mediators, such as NF-kB and tumor necrosis factor (TNF)-α, in-creased Nrf2 expression, normalized ROS and GSH levels, and improved memory def-icits in the OKA-induced model [48]. SFN was able to exert anti-inflammatory effects by reducing astrogliosis in a tauopathy mouse model [49].

A study examined the effects of SFN combined with NAC in vitro using co-cultures of neurons and BV2 cells, and in an in vivo model of traumatic brain injury (TBI). In vitro results showed a more pronounced neuroprotective and antioxidant ef-fect in cells treated with SFN alone, compared to the combination with NAC. Howev-er, in the in vivo model, the combination of SFN and NAC reduced the levels of in-flammatory biomarkers, with a modest improvement in deficits in rats with TBI [50].

A study showed that SFN reduced inflammation not only in the brain but also pe-ripherally. In LPS-treated mice, SFN decreased pro-inflammatory cytokine production in the hippocampus and liver, but did not improve the response to LPS-induced dis-ease [51]. Neuroinflammation can also affect the retina, leading to degeneration that is found in various pathologies, such as retinitis pigmentosa. An in vivo experiment showed that SFN exerted a protective effect on the retina, reducing inflammation, in-flammatory markers, and glial cell activation, thus preventing retinal degeneration associated with these processes [52]. In a hyperammonemia model linked to cognitive impairment in liver cirrhosis and minimal hepatic encephalopathy (MHE), SFN treat-ment shifted macrophages from M1 to M2, reduced hippocampal inflammation, and normalized GAT-3 expression. This decreased GABA levels, restored the glutamate–nitric oxide–cGMP pathway, and improved learning and motor coordination [53]. The activation of NLRP3-mediated inflammasome plays a key role in the neuroinflamma-tion process during acute ischemic stroke. In an in vivo study, treatment with SFN re-duced stroke volume by about 40%, significantly improving functional recovery com-pared to control animals treated with a vehicle, thanks to the ability of SFN to inhibit the activation of NLRP3 [54].

The consumption of SFN-enriched broccoli sprouts improved the neuroinflam-matory process, reducing the activation of NF-κB pathway, cytokine release, apoptosis, and improving memory deficits [55]. It was shown that a diet including 10% broccoli could be useful in reducing this aging-related neuroinflammation [56].

BITC is an ITC found in several cruciferous vegetables, known for its neuroprotec-tive properties. Treatment with BITC reduced the levels of IL-1β and inhibited the ac-tivation of the NLRP3 inflammasome in LPS-induced microglial cells. In addition, it also decreased the production of ROS and the activation of the transcription factor NF-κB [57]. An extract of *Eruca sativa* seed was also able to inhibit NLRP3 in motor neurons stimulated with LPS by counteracting apoptosis and pro-inflammatory cyto-kine production [58]. Jafaaru et al. showed that pre-treatment with glu-comoringin-isothiocyanate (GMG-ITC), an ITC derived from moringa oleifera, reduced ROS production by activating Nrf2, influencing the MAPK pathway, and lowering NF-κB levels in H_2_O_2_-treated cell line [59]. Another ITC that had a neuroprotective ac-tivity is AITC, an aliphatic ITC from *Wasabia japonica* (wasabi). AITC reduced the re-lease of TNF-α, IL-6, PGE2, and NO by inhibiting COX-2 and iNOS production in LPS-induced BV2 cells [60]. The study by Latronico and coworkers evaluated the ef-fects of three ITCs (AITC, PEITC, and SFN) in LPS-induced astrocytes, showing that all three compounds inhibited the release of metalloproteinase (MMP)-2 and MMP-9 [61]. Based on the beneficial properties of ITCs, the compound ITH12674 was synthesized combining melatonin and SFN. ITH12674 provided anti-inflammatory and antioxi-dant effects, activating Nrf2 and increasing GSH levels [62]. Secondarily, ITH12674 improved locomotion and social interactions in animals treated with LPS, showing benefits at both cellular and behavioral levels [63]. Based on the data provided, ITCs may be an effective approach to reducing neuroinflammation and oxidative stress. An overview of these studies is presented in Table 1.

## 5. Neurodegenerative Disorders

### 5.1. Alzheimer’s Disease (AD)

AD is a common neurodegenerative disorder, affecting about 10% of individuals over 80 years old. Despite extensive research, its exact pathogenesis remains unclear [64]. However, it is known that neuronal loss is linked to the formation of senile plaques due to the accumulation of amyloid β (Aβ). Under normal conditions, Aβ has regulatory functions, aids in axonal growth, and influences synaptic plasticity. Another key feature of AD is the hyperphosphorylation of the tau protein, leading to neurofibrillary tangles that disrupt intraneuronal communication [65]. Studies show that the accumulation of Aβ and tau in AD is caused by factors like protein folding dysfunction in the endoplasmic reticulum, increased oxidative stress, and impaired protein elimination through the proteasome and autophagy [66,67]. Currently, there is no treatment able to cure AD or delay the disease progression. The discovery of new natural compounds with novel therapeutic effects is a fast-growing field of interest for the treatment of AD. The intake of foods or plant extracts with antioxidant properties could have beneficial effects on human health and improve brain functions [68].

#### Preclinical Studies on Glucosinolates or Isothiocyanates in AD

SFN is one of the main ITCs studied for the treatment of AD. In an in silico study, it was found that SFN was able to regulate 45 targets involved in different processes implicated in AD pathophysiology, such as inflammation, insulin resistance, and apoptosis [69]. A docking study on two different types of broccoli, Romanesco broccoli (RB) and purple broccoli (PB), revealed that extracts from both varieties exhibit antioxidant and anticholinergic properties [70]. One study revealed, using electrospray ionization mass spectrometry (ESI-MS), that the SFN reduced the aggregation of Aβ_1–40_ [71]. A computational study showed that SFN could be considered as a possible inhibitor of the β-site amyloid precursor protein-cleaving enzyme 1 (BACE1), which is involved in the production and deposition of Aβ. The inhibition of BACE1 could play an important role in AD prevention [72].

In the pathogenesis of AD, oxidative stress is a crucial event leading to neuronal damage; the modulation of Nrf2 expression could be one of the key strategies for treating AD [73]. In Villavicencio-Tejo and colleagues’ study, SFN increased the expression of antioxidant proteins such as catalase, NAD(P)H quinone oxidoreductase (NQO1), and Nrf2, as well as preventing mitochondrial dysfunctions in immortalized cortical neurons transfected with tau protein [74]. There are several molecular mechanisms regulating Nrf2 expression. SFN was able to act at the epigenetic level, increasing the expression of Nrf2 and then enhancing antioxidant and anti-inflammatory capacity in an in vitro model of AD (N2a/APP cells) [75]. Furthermore, SFN showed a neuroprotective effect in a model of nitrosative stress induced by sodium nitroprusside (SNP) [76]. Impairment of the autophagic process leads to the accumulation of Aβ aggregates. SFN was able to enhance the phagocytosis in microglial cells exposed to Aβ, helping to remove Aβ aggregates via the reduction in inflammation [77]. Moreover, SFN reduced Aβ and tau accumulation by increasing the levels of heat shock protein (HSP) 70 and the chaperone protein CHIP. This decreased plaque formation, inhibited apoptosis, and alleviated memory deficits [78,79]. SFN increased the expression of a specific water channel protein, aquaporin-4 (AQP4), via the p38 MAPK pathway [80]. SFN can also restore the levels of MerTK, a protein involved in the process of Aβ phagocytosis. Downregulation of MerTK contributes to chronic inflammation, a key process in AD. Restoring its function helps improve amyloid removal and reduce inflammation, thus counteracting the disease [81]. These results may suggest how SFN modulates the clearance of Aβ, tau, and other soluble proteins.

The activation of inflammasome is involved in the Aβ-induced inflammation process. One study showed that SFN inhibited NLRP3 in Aβ-activated microglial cells, reducing pro-inflammatory cytokine release, ROS production, and cytostatic autophagy [82]. In addition, SFN could reverse the M2 phenotype to an M1-like phenotype in activated BV-2 cells. This process reduced inflammation by downregulating the MAPK/NF-κB signaling pathway. Typically, M2 microglia are involved in anti-inflammatory and tissue repair functions, whereas M1 microglia are activated in response to stimuli like Aβ, leading to a pro-inflammatory response [83].

The in vitro results were also replicated in in vivo models. SFN exerted neuroprotection via the increase in BDNF expression and key proteins involved in neuronal plasticity and memory, such as TrkB and p-CREB [84]. In addition, SFN was able to inhibit apoptosis and oxidative stress by reducing MAPK activation (ERK1/2, JNK, and p38) and increasing GSH levels in a model induced by MGO, a glycation product that is present at high levels in the cerebrospinal fluid of AD patients [85]. SFN in different AD models ameliorated spatial cognitive impairment, reduced Aβ deposition [86], and regulated specific histone deacetylases (HDACs), resulting in diminished plaque accumulation [87]. In addition, SFN was able to reduce the deposition of Aβ and improve behavioral deficits in an AD model, where disease-like lesions were induced by the combined administration of D-galactose and aluminum [88]. SFN inhibited tau protein phosphorylation in an in vivo model of AD via the PI3K/Akt/GSK-3β pathway and suppressed anti-inflammatory markers such as NF-κB, NO, TNF-α, and IL-6 [89]. In addition, it inhibited cathepsin B and caspase-1 production, decreasing IL-1β release by blocking Aβ-induced inflammasome [90]. It also reduced oxidative stress, enhanced mitochondrial function in the brain, and improved cognitive impairments seen in both AD and type 2 Diabetes Mellitus, where oxidative stress plays a key role in disease progression and raises the risk of developing AD [91]. In addition, thanks to the activation of Nrf2, SFN mitigated cognitive decline and hippocampal AD-like lesions in diabetic mice [92]. SFN was also able to ameliorate cognitive vascular impartment (CVI) through the reduction in Aβ and p-tau accumulation [93]. Furthermore, SFN was able to inhibit apoptosis and Aβ-induced DNA damage. This is possible through its action on the PI3K-Akt signaling pathway and the BRCA1 gene, which plays an important role in DNA protection [94]. SFN also reduced the loss of cholinergic neurons [95], improved neurobehavioral disorders, and exerted anxiolytic effects [96].

The LPS model was used to investigate the link between neuroinflammation and AD. Studies showed that systemic LPS administration in vivo leads to behavioral changes such as learning and memory deficits, reduced appetite, decreased movement, and weight loss, which are clinically associated with neurodegenerative diseases in humans [97]. In addition, consecutive administration of LPS in mice could potentially increase the production and aggregation of Aβ in the cerebral cortex [98]. Moreover, Alzharani et al. found that SFN pre-treatment in an LPS model improved memory and reduced oxidative stress, Aβ accumulation, and auto cells activation. These effects were mediated through the AMPK signaling pathway, which resulted in decreased caspase-3 expression [99]. Several studies have been conducted on the effects of SFN on cognitive impairment associated with AD. Ho Sub et al. used an in vivo scopolamine (SCOP) model to assess the effect of SFN on memory deficits. SFN treatment improved memory performance, reversing SCOP-induced declines in short-term and long-term memory, and increased BDNF and CREB levels, enhancing hippocampal activity [100]. In addition, SCOP was used in an in vivo model of zebrafish. In this model, SFN was able to enhance the cognitive function in zebrafish inducted by SCOP through the attenuation of cholinergic neural loss [101]. Furthermore, SFN protected the cerebral vascular system during inflammation by activating Nrf2, blocking NF-κB, and downregulating E-cadherin and VCAM. This also helps reduce the recruitment of pathogenic leukocytes to the brain [102]. The antioxidant effect of broccoli ITC extract was tested in Caenorhabditis elegans. Hortal and colleagues showed in C. elegans that the broccoli extract (BRO) was able to reduce the concentration of ROS through induction of the transcriptional factor skn-1/Nrf2 and its downstream genes gst-4 and hsps. In addition, BRO reduced Aβ-induced paralysis [103]. Another ITC tested in in vitro and in vivo models is 6-methylsulfinyl hexyl isothiocyanate (6-MSITC). 6-MSITC improved memory deficits in Aβ_1–42_-induced mice by activating Nrf2, which increased disintegrin A and metalloproteinase 17 (ADAM17) levels, a key factor in synaptic function [104]. It also reduced cognitive impairment in an AD mouse model by suppressing oxidative stress, apoptosis, and neuroinflammation [105,106]. MOR, extracted from *Moringa oleifera*, is considered a potential treatment for AD. *Moringa oleifera*, also known as the “miracle tree,” is a member of the Moringaceae family, which includes 12 other species [107]. MOR reduced the expression of genes involved in different processes such as senescence, autophagy, and mitophagy, and mitigated the cytotoxicity induced by Aβ by decreasing caspase expression [108]. Additionally, pre-treatment with MOR was also able to inhibit the gene involved in mitophagy in stem cells of the human periodontal ligament. In neurodegenerative disease, stem cell-based therapeutic intervention offered an alternative approach meant to repair the damaged tissue [109]. ITCs are considered as H_2_S donors. Based on this property, a synthetic compound was developed and tested in vitro, obtained by combining the ITCs with memantine, also called Memit. The study showed the neuroprotective effects of Memit in LPS-treated cells, demonstrating a reduction in ROS levels and an inhibition of Aβ aggregation [110]. In addition, other H_2_S-releasing compounds have been synthesized by combining rivastigmine, an acetylcholinesterase (AChE) inhibitor with two ITCs, SFN and ER, an ITC from *Eruca sativa* Mill. The results showed that these new compounds had anti-inflammatory and antioxidant action in LPS-induced microglia cells. In addition, the compounds exerted neuroprotective effects against Aβ-induced toxicity in SH-SY5Y cells [111]. From the collection of in vitro and in vivo data, although further studies and clinical approaches are needed, the use of ITCs could represent a new approach for the treatment of AD. An overview of the described studies is presented in Table 2.

### 5.2. Parkinson’s Disease (PD)

PD is a neurodegenerative disorder characterized by both motor symptoms—such as bradykinesia, rigidity, postural instability, tremor—and non-motor symptoms, including autonomic dysfunction, cognitive issues, pain, sleep, and mood disorders. One cause of neurodegeneration is the loss of dopaminergic neurons in the substantia nigra and other brain regions. The disease is also linked to the accumulation of Lewy bodies (LBs), cytoplasmic inclusions primarily composed by the protein α-synuclein (α-syn), which leads to neuronal death [64]. The most common treatment for PD is L-DOPA, a dopamine precursor that compensates for dopamine deficiency but does not arrest disease progression. Additionally, many patients do not respond to dopaminergic therapy, and L-DOPA may increase ROS, further contributing to neurodegeneration [112]. Phytochemicals present in plant-based foods, including ITCs, may provide an improvement in symptomology and disease progression [113].

#### 5.2.1. Preclinical Studies on Glucosinolate or Isothiocyanates in PD

A study on red cabbage extracts showed neuroprotective effects in an in vitro model of α-Syn-induced PD [114]. One study identified SFN as a potent H_2_S donor among 15 compounds derived from cruciferous vegetables. Due to its ability to release H_2_S, SFN has been tested in an in vivo model of PD, showing improvements in motor skills, a reduction in dopaminergic neuron apoptosis, as well as anti-inflammatory and antioxidant effects [115]. In accordance with a 1-methyl-4-phenyl-1,2,3,6-tetrahydropyridine (MPTP)-induced PD model, SFN was able to reduce the production of ROS through the modulation of the Nrf2 pathway [116,117]. Yaoyu Pu and colleagues also demonstrated that the dietary intake of GRA, the precursor of SFN, increased Nrf2 levels and antioxidant enzymes such as HO-1 and NQO1 in MPTP-induced mice. GRA also demonstrated a neuroprotective effect against MPTP-induced dopaminergic toxicity in the brain. These findings suggested that consuming cruciferous vegetables may help prevent PD [118]. Also, chronic oral rotenone administration induces key PD features, such as selective dopaminergic neurodegeneration, abnormal behaviors, and Lewy body formation. Zhou’s study showed that rotenone decreased the expression of Nrf2, HO-1, and NQO1 in the cortex and striatum, while SFN treatment increased these genes and reduced oxidative stress [119]. SFN also provided neuroprotection against rotenone-induced toxicity by modulating autophagy [119]. An in vitro and in vivo study showed that SFN and GRA through Nrf2 activation inhibited an age-dependent transcription factor CCAAT/enhancer-binding protein β (C/EBPβ). This factor is a key factor involved in the regulation of α-Syn expression in PD and aging brains and is also implicated in the neuroinflammatory process [120].

Treatment with cypermethrin, a synthetic pesticide, can damage mitochondrial function and cause the overexpression and aggregation of α-Syn, a typical PD feature. In this model, pretreatment with SFN, both in vitro and in vivo, reduced α-Syn aggregation, oxidative stress, and death of dopaminergic neurons [121].

Methyl-binding protein CpG2 (MeCP2) is a transcriptional repressor that blocks the transcription of BDNF and mutations in its gene cause Rett syndrome (RTT). RTT is classified into typical and atypical forms. RTT patients normally show normal development in the first 6 to 18 months of life, followed by a rapid regression with severe intellectual disability, motor issues, and autism-like symptoms. RTT symptoms include deceleration in head growth, loss of purposeful hand movements, abnormal gait, repetitive hand-wringing motions, loss of language function, breathing irregularities, and cognitive impairments. Some atypical cases of RTT present mutations in cyclin-dependent kinase-like 5 (CDKL5). The diagnostic criteria for atypical RTT include the presence of regression associated with 2 or more of the main criteria for classic RTT (loss of acquired purposeful hand skills, loss of acquired spoken language, gait abnormalities, stereotypic hand movements), a period of regression followed by recovery or stabilization, and 5 out of 11 supportive criteria (breathing difficulties, bruxism, impaired sleep pattern, abnormal muscle tone, peripheral vasomotor disturbances, scoliosis/kyphosis, delayed growth, small cold hands and feet, inappropriate laughter or screaming spells, decreased pain sensation, and intense eye communication) [122]. The preserved speech variant is a milder RTT form, in which patients show the same stages of this condition and make slow progress in manual and verbal abilities [123]. RTT patients with mutations in the MeCP2 show motor deficits in association with intellectual disability. Thus, it was supposed that MeCP2 may also be involved in PD pathogenesis. An in vitro and in vivo study showed that SFN was able to exert a neuroprotective effect by increasing the expression of BDNF and suppressing the expression of MeCP2. This result showed that MeCP2 could be a new therapeutic target [124].

Additionally, SFN was able to decrease apoptosis in in vitro and in vivo PD models by the modulation of the mTOR pathway [119]. A study compared the effects of ER and SFN. The biotransformation mechanisms of both compounds were similar [125]. Both ER and SFN enhanced Nrf2 and GSH expression, reduced ROS, and prevented apoptosis in a 6-hydroxydopamine (6-OHDA)-induced SH-SY5Y model. In addition, in vivo, both compounds enhanced motility, reduced damage to dopaminergic neurons, and increased levels of tyrosine hydroxylase (TH), a marker of dopaminergic function. They also reduced DNA fragmentation and neuronal death, confirming the beneficial effects observed in in vitro studies [126]. The neuroprotection ability of ER was also tested in the C. elegans model. The results showed that ER reduced α-Syn aggregation and restored motor deficits [127]. Besides ER, the neuroprotective effects of GMG were also evaluated in PD models. In a study by Giacoppo et al., the effects of GMG and its bioactivated form, MOR, were compared. The results revealed that MOR was more effective than GMG in reducing the production of pro-inflammatory cytokines, such as TNF-α and IL-1β, and in lowering the expression of the TLR4 receptor, which plays a role in the release of inflammatory mediators in LPS-stimulated RAW 264.7 macrophages. In vivo results showed that MOR had a stronger neuroprotective effect than GMG, reducing MPTP-induced toxicity at the dopaminergic level. Behavioral tests revealed improved motor coordination and reduced bradykinesia in mice treated with MOR compared to those treated with GMG. Additionally, MOR pre-treatment preserved neuronal cell integrity and enhanced synaptic communication. MPTP mice pre-treated with MOR showed reduced expression of cleaved caspase-9, p53, and STAT1 proteins, which are involved in apoptosis, compared to those treated with GMG. Both in vitro and in vivo data suggested that MOR may be a promising candidate for PD treatment due to its ability to modulate various molecular pathways [128].

Synthetic ITCs were evaluated in in vitro and in vivo models. ITC-3 reduced cytotoxicity induced by MPTP and BH4 by increasing Nrf2 levels and boosting oxidative stress-related proteins such as GCLC, GCLM, and HO-1. In vivo results showed that ITC-3 can suppress microglial activation, protect dopaminergic neurons from degeneration, and reduce motor deficits associated with PD [129]. Moreover, another synthetic ITC, ITC-57, produced similar results in vitro and in vivo [130]. Therefore, it is evident from the data that the application of synthetic ITCs could be a novel approach for PD treatment.

Oxidative stress plays a crucial role in the pathogenesis of PD. Among the main defense mechanisms, there is GST, a natural antioxidant that protects the brain from oxidative stress and neurodegeneration. In a model of zinc-induced neurodegeneration, it was observed that it inhibited the expression of GST-π, increasing oxidative stress and contributing to the degeneration of dopaminergic neurons. However, one study showed that pre-treatment with BITC can reverse the negative effects of zinc by increasing levels of GST-π, improving behavioral disorders and reducing both oxidative stress and apoptosis markers [131]. A study by Morroni et al. showed the neuroprotective effects of 6-MSITC. Morroni et al. demonstrated that in an in vivo PD model induced by 6-OHDA, preventive treatment with 6-MSITC reduced 6-OHDA-induced neurotoxicity in mice. Additionally, 6-MSITC decreased oxidative stress and apoptosis, while improving behavioral disorders, particularly motor deficits [132]. An overview of the described studies is presented in Table 3.

#### 5.2.2. Clinical Studies on Glucosinolates or Isothiocyanates in PD

A clinical trial was conducted on PD patients to evaluate the efficacy of broccoli seed tea (BST), enriched with SFN, in inducing Nrf2 gene activation. The case study involved 17 patients, who were given a special BST for four weeks, containing a daily dose of 25–40 μmol of SFN (once per week during the first week with a possibility to increase to twice per week from the second week). A new Redox Stress Test technique was used to analyze the effects of the treatment, identifying symptoms related to oxidative stress, a major cause of disorders in PD, and observing the impact of Nrf2 pathway activation on these symptoms. Although the results did not show precise statistical values (*p*-value), thus making statistical significance uncertain, it was found that the BST treatment improved non-motor symptoms such as fatigue, constipation, and urinary urgency, but without affecting motor symptoms. These preliminary results suggested that oxidative stress plays a crucial role in the onset of non-motor symptoms, both in the central nervous system and peripheral organs. A redox imbalance, in fact, is responsible for progressive neurological damage in the brain, which initially is not easily detectable. Specific neurological symptoms do not manifest themselves until many years later, when dopamine deficiency causes impairment of motor control, leading to the onset of a movement disorder typical of PD [133]. Actually, another randomized placebo-controlled phase 2 trial (NCT05084365) is currently underway to test the effects of SFN on 100 PD patients.

### 5.3. Multiple Sclerosis (MS)

MS is an immune-mediated inflammatory disorder of the CNS, affecting over 2 million people globally [134]. The disease onset is driven by the loss of blood–brain barrier (BBB) integrity, providing T lymphocytes to cross. Once activated, T cells initiate demyelination, reactive gliosis, and neuronal degeneration [134]. Neurodegeneration, oxidative stress, and neuroinflammation play a main role in the progression of MS. Indeed, ROS production, due to an impaired antioxidative system, triggers the release of pro-inflammatory cytokines, which worsen the pathology [135]. Oligodendrocytes appear to be particularly susceptible to oxidative stress and to cytokines, which can induce their death, inhibiting differentiation and altering demyelination processes [136]. New neuroprotective therapies are needed to reduce long-term neurological disability, and the use of natural compounds with antioxidant properties could represent a potential protective approach.

#### Preclinical Studies on Glucosinolates or Isothiocyanates in MS

An in vitro study on oligodendrocytes showed that SFN exerted a neuroprotective action reducing the production of ROS and promoting differentiation [137]. In accordance with the main role of inflammation in MS, recent studies showed that the macrophage migration inhibitory factor (MIF) represents a key factor in several autoimmune diseases, including MS, where it is found to be overregulated. One study showed that some synthetic ITCs, particularly those with an aromatic structure, were able to inhibit the activity of MIF. This mechanism could open up new therapeutic prospects for the treatment of inflammatory diseases [138]. Given the complexity of the disease, to date, the majority of the studies investigating the protective effects of phytocompounds in MS were carried out in in vivo models. One of the most commonly used in vivo models for studying MS is the experimental autoimmune encephalitis (EAE) model. It is characterized by the perivascular infiltration of inflammatory cells, leading to demyelination in the brain and spinal cord through an autoimmune cascade mediated by CD4+ T cells [139]. SFN is one of the phytocompound tested in MS in in vivo models. SFN can cross the BBB, a property that has paved the way for its potential use in the treatment of MS. Yoo and colleagues, in their study, showed that SFN treatment reduced iNOS levels, inhibited inflammatory cell infiltration, and prevented demyelination in the spinal cord of EAE mice [140]. In addition, bioactive GRA was observed to exert neuroprotective effects in an experimental in vivo model of EAE. The study showed that bioactive GRA was able to prevent cell death by reducing caspase-3 levels, counteracting the BBB dysfunction through the increase in the expression of tight junction proteins and modulating genes involved in inflammatory pathways [141]. Galea et al. evaluated the effects of synthetic SFN SFX-01 in a PLP 139–151-induced EAE model. The research assessed the use of SFX-01 for both therapeutic and preventive purposes. Histological analysis of the spinal cord showed improved demyelination, reduced apoptotic cells, and decreased symptom severity [142]. These results suggested that SFN could be used as an integrative therapy in the treatment of MS due to its anti-inflammatory properties. In addition to SFN, GMG-ITC was also tested in vivo for the treatment of MS. Galuppo and collaborators showed that GMG-ITC treatment reduced lymphocyte infiltration, demyelination, and axonal loss in MOG mice. It also modulated neuroinflammation by affecting the MAPK pathway, lowering TNF-α levels, and decreasing ROS production [143]. In addition, GMG-ITC exerted anti-inflammatory effects by normalizing the Wnt-beta catenin pathway and inhibiting GSK3B, which blocked the release of inflammation mediators like IL-1β, IL-6, and COX-2 [144]. Additionally, Giacoppo et al. showed that GMG-ITC provided neuroprotection through the modulation of apoptosis-related genes and increased Nrf2 expression, demonstrating antioxidant properties [144]. In another study, Giacoppo and coworkers demonstrated that the topical application of a MOR-based cream helped relieve neuropathic pain associated with MS. While the exact causes of neuropathic pain are not fully understood, it is believed to involve inflammatory processes and ion channel dysfunction. Treatment with MOR-based cream reduced pro-inflammatory cytokines (TNF-α, IFN-γ) and increased levels of the anti-inflammatory cytokine IL-10. Additionally, it provided neuroprotective effects by blocking ion channels [145]. The data collected suggested that the use of ITCs could represent a new approach to the treatment of the disease. An overview of the described studies is presented in Table 4.

## 6. Neurodevelopmental Disorders, Psychiatric Conditions, and Epilepsy

Recent clinical and preclinical studies explored the use of GLS and their derivates as potential treatments for neurodevelopmental conditions, psychiatric disorders, and epilepsy. These studies represent the first attempts to address disorders of heterogeneous origin and pathophysiology by targeting a limited number of common pathways through the use of natural compounds such as ITCs and GLS. Preclinical evidence suggests that GLS and their metabolites, particularly SFN, possess various biological properties relevant to neurological and psychiatric conditions [146,147]. However, it remains unclear whether there are key mechanisms shared across multiple disorders or if these mechanisms are specific to each condition. Supported by mechanistic data, recent preclinical and clinical studies were developed to explore the use of SFN in treating autism, psychiatric conditions such as depression and schizophrenia, and epilepsy.

### 6.1. Autism Spectrum Disorder (ASD)

ASD is a neurodevelopmental disorder characterized by deficits in communication and social interaction, as well as repetitive behaviors or activities [148]. To date, the etiopathogenesis of ASD remains unknown. According to estimates from the CDC’s Autism and Developmental Disabilities Monitoring (ADDM) Network, ASD affects approximately 1 in 36 children and is four times more common in boys than in girls [149]. Currently, no pharmacological therapy has been approved to reduce the core symptoms or address the pathophysiological processes associated with ASD [150]. GLS and their derivates have gained increasing interest for their potential therapeutic effects in ASD, thanks to their ability to modulate key pathophysiological pathways, including oxidative stress, inflammation, and gut microbiota regulation.

#### 6.1.1. Preclinical Studies on Glucosinolate or Isothiocyanates in ASD

Preclinical studies were conducted to elucidate the molecular mechanisms by which SFN alleviates autism-like symptoms. A key mechanism of SFN is the activation of Nrf2, a transcription factor regulating the expression of genes involved in antioxidant defense and inflammatory response. At the cellular level, SFN restored alterations in the thioredoxin 1/thioredoxin reductase 1 (Trx1/TrxR1) system in neutrophils of individuals with ASD, a crucial process for redox balance. Notably, exposure to methylmercury, an environmental contaminant associated with increased ASD risk, inhibited TrxR1 activity and increased ROS production in neutrophils of ASD subjects. SFN reversed these effects via Nrf2 activation [151]. Encapsulation of SFN in extracellular vesicles (EVs) from amniotic fluid (SFN@EVs) enhanced its stability and targeted delivery, improving its bioavailability. SFN@EVs protected hPC-12 cells from oxidative stress by activating the Nrf2 pathway, which in turn reduces the expression of IL-6, a key pro-inflammatory cytokine linked to ASD [152]. Additionally, studies in chick embryos exposed to sodium valproate (an ASD model) confirmed the protective role of SFN@EVs, improving embryonic survival and preserving normal gray and white matter structure in the brain [152]. SFN also modulated immune responses. In peripheral blood mononuclear cells (PBMCs) from healthy donors, SFN treatment increased the expression of cytoprotective enzymes such as NQO1, HO-1, and AKR1C1 while suppressing LPS-induced inflammation by lowering IL-6, IL-1β, COX-2, and TNF-α levels. Similar effects were confirmed in PBMCs from ASD children treated with SFN for 14 days (NCT02561481) [153].

Monocytes from ASD subjects showed a reduced expression and activity of Nrf2, leading to increased NF-κB activation and overexpression of IL-6, IL-1β, iNOS, and nitrotyrosine, markers of inflammation and nitrative stress. SFN treatment restored Nrf2 activity, blocking NF-κB activation and reducing inflammation and oxidative stress in monocytes from ASD subjects. It also upregulated antioxidant enzymes, such as SOD1, GPx1, and GR, enhancing cellular defenses [154]. In BTBR T + Itpr3tf/J (BTBR) mice, a widely used ASD model, SFN improved social interaction and reduced repetitive behaviors such as self-grooming and marble burying. These beneficial effects correlated with reduced Th17 immune responses, as indicated by lower expression of STAT3, RORC, IL-17A, and IL-23R in CD4+ T cells. SFN also reduced oxidative stress markers (NF-κB, iNOS, and lipid peroxidation) in the cerebellum and neutrophils [155]. In BTBR and C57BL/6 (C57) mice, SFN treatment enhanced enzymatic antioxidant defenses in neutrophils and the cerebellum by increasing SOD, GPx, and GR activity, suggesting a neuroprotective effect of SFN through redox balance modulation [155]. In a maternal immune activation (MIA) ASD model, SFN not only improved social deficits but also altered gut microbiota composition, particularly affecting Bacillales, Staphylococcaceae, and Hamophilus families, suggesting a possible microbiota–brain interaction [156]. Furthermore, GRA-derived SFN demonstrated protective effects against MIA-induced ASD. Maternal dietary supplementation with 0.1% GRA during pregnancy and lactation prevents cognitive and social impairments in juvenile offspring. In adult MIA-exposed mice, a GRA-enriched diet restored parvalbumin immunoreactivity in the medial prefrontal cortex (mPFC), a biomarker of neurodevelopmental disorders [157]. These findings highlight SFN as a promising nutritional and therapeutic strategy for mitigating oxidative stress, neuroinflammation, and behavioral abnormalities in ASD, offering potential benefits for both children and at-risk populations. An overview of the described studies is presented in Table 5.

#### 6.1.2. Clinical Studies on Glucosinolate or Isothiocyanates and ASD

The first evidence of the beneficial effects of SFN in individuals with ASD emerged from a double-blind, randomized, placebo-controlled trial conducted at Massachusetts General Hospital (NCT01474993) between 2011 and 2013 [158]. The study aimed to improve core ASD symptoms and underlying biochemical abnormalities, such as oxidative stress, antioxidant deficiency, and inflammation, through SFN administration. Male participants aged 13 to 27 years with moderate-to-severe ASD received daily capsules containing SFN derived from broccoli sprouts (50–150 µmoL; approximately 1.4 µmoL/kg/day) for 18 weeks, followed by a 4-week washout period. SFN treatment led to significant and reversible improvements in social interaction, abnormal behavior, and verbal communication in treated subjects (*n* = 29) compared to the placebo group (*n* = 15). However, these effects diminished after the washout period, indicating the treatment’s transient nature. SFN exhibited a favorable safety profile, with only minor adverse effects such as gastrointestinal discomfort, fever, seasonal allergies, irritability, and cough. Following this trial, a subset of responders (*n* = 10) continued SFN-based dietary supplements under caregiver supervision to maintain the observed behavioral improvements. These individuals were periodically monitored over three years, with many continuing to report sustained benefits [159]. This clinical success prompted the development of five additional trials investigating SFN’s effects in ASD (NCT02561481, NCT02677051, NCT02909959, NCT02654743, and NCT02879110), some of which have since been completed. The first of these studies (NCT02654743), led by Robert Hendren, was an open-label study designed to explore associations between urinary metabolites and clinical improvements. Metabolomic analysis of 15 school-aged children with ASD identified 77 urinary metabolites correlated with symptom reduction following daily SFN supplementation with GRA and the enzyme MYR. These urinary metabolites were linked to oxidative stress, amino acid metabolism, gut microbiome-related amino acid metabolism, neurotransmitter metabolism, stress response hormones, and sphingomyelin metabolism [160]. A subsequent randomized, double-blind, placebo-controlled clinical trial involving 60 children with ASD (aged 4 to 12 years) evaluated SFN in combination with risperidone, a standard treatment for irritability in ASD. Participants were assessed using the Aberrant Behavior Checklist (ABC)—Community Edition at weeks 5 and 10. Significant improvements were observed in the Irritability and Hyperactivity/Noncompliance subscales in the SFN-treated group compared to the placebo group, with a significant effect over time for both symptoms. However, no significant changes were observed in other behavioral domains, including lethargy/social withdrawal, stereotypic behavior, or inappropriate speech. SFN treatment was well tolerated, with no increase in adverse effects, suggesting its potential as an adjunctive treatment for managing irritability and hyperactivity in ASD [161]. Building on these findings, a randomized controlled clinical trial (NCT02561481) was conducted to further investigate SFN’s therapeutic potential in ASD [162]. This study enrolled 45 children with ASD (aged 3 to 12 years), who were randomized to receive either GRA + MYR or placebo for 15 weeks, followed by a 15-week open-label phase and a 6-week no-treatment period. Although no statistically significant differences were found in the primary outcome measure evaluating symptom severity, caregivers reported a significant improvement in aberrant behavior (secondary outcome) in the GRA + MYR group compared to placebo. Additionally, significant biomarker changes were detected, including reduced inflammatory cytokines (IL-6, TNF-α) and improved oxidative stress markers. Treatment was well tolerated, with minor side effects such as insomnia, irritability, and abdominal discomfort in some children. While these results highlight SFN’s potential benefits, further research is needed to confirm its long-term efficacy. More recently, a prospective, double-blind, randomized, placebo-controlled study examined the potential benefits of SFN on behavioral and cognitive symptoms in children with ASD (aged 3 to 7 years) [163]. Despite a 36-week treatment period (50 µmol of SFN daily), no statistically significant differences were observed between the SFN and placebo groups across multiple assessment scales, including the Autism Diagnostic Observation Schedule-2 (ADOS-2), the Social Responsiveness Scale-2 (SRS-2), and the ABC. These findings raise questions about SFN’s efficacy in younger children with ASD and highlight the need for larger, longer-term studies to account for individual response variability and placebo effects. Magner et al. [163] concluded that while SFN possesses antioxidant and neuroprotective properties, current evidence does not support its use in preschool-aged children with ASD.

A longitudinal clinical study explored SFN’s impact on gut microbiota, an emerging factor in ASD pathophysiology. Children treated with GRA + MYR for 12 weeks showed significant improvements in verbal and non-verbal communication, as assessed by the OSU Autism Rating Scale-DSM-IV (OARS-4). However, microbiota alterations in treated subjects were less pronounced than those reported in animal models [156], although correlations were found between specific bacterial taxa and behavioral improvements [156]. In a multicenter, randomized, double-blind, placebo-controlled trial, SFN’s efficacy in children with ASD was further evaluated [164]. The study enrolled 108 children (aged 3–15 years), revealing significant improvements in clinician-rated assessment scales, with 30% of SFN-treated participants exhibiting a ≥30% symptom reduction after 12 weeks. However, no significant differences emerged in caregiver-reported evaluations. The benefits of SFN appeared more pronounced in children over the age of 10, suggesting age-related differences in treatment responsiveness. These discrepancies between clinician and caregiver assessments underscore the need for further studies to refine dosing strategies and establish SFN’s clinical utility. Despite promising evidence suggesting SFN’s role in redox regulation, inflammation, and microbiota modulation in ASD, clinical findings remain inconsistent. While some trials report significant behavioral and communication improvements, others do not find statistically meaningful effects. This variability may stem from differences in study design, treatment duration, dosage, participant age, and SFN bioavailability. Additionally, SFN’s precise mechanisms of action in gut microbiota modulation and neuroinflammation remain unclear. Thus, further large-scale clinical studies are essential to confirm SFN’s efficacy, optimize dosing regimens, and develop personalized therapeutic strategies for individuals with ASD. An overview of the described studies is presented in Table 6. Recent studies explored the effect of SFN on specific genetic polymorphisms, such as those of the COMT and CBS genes, which are involved in the metabolism of sulfur-containing compounds. In particular, it has been found that some autistic patients with homozygous genotypes (rs4633-TT and rs4680-AA) in COMT variants exhibit a more pronounced response to SFN intake, whereas others with heterozygous genotypes (rs4633-CT and rs4680-AG) may metabolize the compound differently. These findings suggest that the effectiveness of GLS metabolites may vary depending on individual genetic variants, highlighting the importance of personalized nutritional treatment based on the patient’s genetic profile [165].

### 6.2. Schizophrenia

Schizophrenia is a progressive neurodevelopmental disorder marked by positive symptoms (hallucinations, delusions) and negative symptoms (e.g., apathy, reduced ability to experience pleasure) that severely impairs cognitive functions, perception, decision-making ability, and emotional regulation, profoundly affecting daily life and interpersonal relationships [166,167]. The etiology of schizophrenia is complex, with a strong genetic component. Unlike disorders such as ASD, schizophrenia is generally treated with high doses of antipsychotic medications that, although effective in controlling symptoms, can cause significant long-term side effects. In recent years, research has focused on the early identification of the prodromal phase of schizophrenia and the adoption of preventive intervention strategies. Since cognitive impairment is often a prodromal symptom, early intervention at this stage could significantly reduce the risk of psychopathological progression into adulthood [168,169]. In this context, increasing attention has been directed toward the integration of ITCs and GLS, powerful natural antioxidants, into the diet. These compounds, due to their neuroprotective effects and ability to modulate oxidative stress—a key factor in the pathogenesis of schizophrenia—could represent a promising therapeutic strategy for the treatment and prevention of cognitive deficits in this condition.

#### 6.2.1. Preclinical Studies on Glucosinolates or Isothiocyanates in Schizophrenia

Several studies highlighted the involvement of neuroinflammatory processes in schizophrenia, with a particular focus on the aberrant activation of microglia, the brain’s resident immune cells. SFN exhibited neuroprotective properties by modulating microglial activity and reducing oxidative stress in hiPSC-derived microglia-like cells (iMGLC). It enhanced microglial phagocytosis after 24 h of treatment and exerted anti-inflammatory and antioxidant effects by activating the Nrf2 pathway.

Specifically, SFN increased GCLM and HMOX1 expression in control iMGLC and healthy twin iMGLC, but not in iMGLC derived from twins affected by schizophrenia. It also downregulated FOS, a marker of the pro-inflammatory NF-κB pathway, only in control cells. These findings suggested that SFN can enhance microglial function but has a variable response in schizophrenia, indicating potential differences in microglial reactivity [170]. Preclinical studies conducted on animal models highlighted the neuroprotective potential of GRA and its derivative SFN, in cognitive deficits associated with schizophrenia. In a study on mice, prophylactic treatment with SFN, a potent activator of the transcription factor Nrf2, showed beneficial effects against phencyclidine-induced cognitive deficits. This was achieved by reducing the increase in 8-oxo-dG-positive cells (a marker of oxidative DNA damage) and preventing the decrease in parvalbumin-positive cells (GABAergic inhibitory neurons that express parvalbumin and play a role in cognitive impairment in schizophrenia) in mPFC and hippocampus. Moreover, phencyclidine-induced cognitive deficits were improved by subsequent therapeutic treatment with SFN [171]. Interestingly, dietary intake of SFN’s precursor, GRA, during childhood and adolescence prevented the onset of cognitive deficits, the increase in 8-oxo-dG-positive cells, and the reduction in parvalbumin-positive cells in the brain (mPFC and CA1) induced by phencyclidine in adult mice. Genetic studies also revealed an epistatic interaction between the NRF2 and KEAP1 genes, influencing cognitive abilities in schizophrenic patients. These findings suggest that dietary intake of SFN or its precursors during childhood and adolescence may prevent the onset of psychosis in adulthood [171].

MIA during pregnancy has been associated with cognitive deficits and reduced parvalbumin immunoreactivity in the mPFC of adult offspring—alterations characteristic of schizophrenia. However, preclinical studies in mice demonstrated that dietary supplementation with GRA during childhood and adolescence can prevent these deficits by improving gene expression in key brain regions, such as the prefrontal cortex and hippocampus. Specifically, GRA regulated the expression of the suppressor of fermentation-induced loss of stress resistance protein 1 (Sf1) gene, whose altered function has been observed in post-mortem brain tissue and hair follicles of schizophrenia patients. Gene expression analysis revealed that MIA disrupts the activity of genes related to the centrosome (which is involved in various cellular processes, including migration, division, and differentiation). Notably, GRA supplementation was able to normalize these genetic patterns, suggesting a possible role of these genes in the pathogenesis of psychosis.

These findings indicate that consuming GRA-rich foods, such as broccoli sprouts, may have a preventive and therapeutic role in cognitive disorders associated with schizophrenia [172]. ITCs showed promising potential in mitigating the adverse effects of antipsychotic drugs. SFN demonstrated significant hepatoprotective and metabolic benefits against olanzapine-induced liver injury, particularly in the presence of a high-fat diet (HFD). In female C57BL/6J mice, SFN reduced hepatic fat accumulation, improved insulin resistance, and lowered plasma transaminases (ALT and AST), key markers of liver damage. Additionally, it alleviated oxidative stress by reducing 4-hydroxynonenal (4-HNE) adducts, suggesting that Nrf2 pathway activation could be a valuable strategy to counteract olanzapine-induced metabolic dysfunction, especially in obesity [173]. Similarly, AITC exhibited protective effects in female BALB/c mice, preventing olanzapine-induced weight gain and adiposity after six weeks of treatment. AITC improved glucose and lipid metabolism by lowering fasting blood glucose, enhancing glucose tolerance, and reducing insulin resistance—effects comparable to metformin. Furthermore, it mitigated inflammation by downregulating NF-κB, TNF-α, and IL-6 and reduced hepatic lipid accumulation by modulating lipogenic and lipolytic gene expression. At the hypothalamic level, AITC restored appetite regulation by counteracting the upregulation of orexigenic neuropeptides (AgRP and NPY), reinforcing its potential as a metabolic protective agent in antipsychotic treatment [174]. In male Wistar rats, SFN further demonstrated its efficacy by reducing weight gain, BMI, and food intake while improving blood pressure and lipid profile (increasing HDL and reducing LDL, VLDL, triglycerides, and total cholesterol). Additionally, SFN alleviated oxidative stress by enhancing antioxidant enzyme activity (SOD, catalase) and reducing lipid peroxidation (MDA) and nitrite levels. At the hepatic level, it suppressed pro-inflammatory cytokines (TNF-α, IL-6, NF-κB) and improved liver function markers (AST, ALT, TBIL), while modulating key metabolic and inflammatory pathways [175]. Together, these findings highlight the therapeutic potential of ITCs, particularly SFN and AITC, in alleviating the metabolic and hepatotoxic side effects of olanzapine, supporting their role as promising adjunct therapies in antipsychotic-induced metabolic disorders. An overview of the described studies is presented in Table 7.

#### 6.2.2. Clinical Studies on Glucosinolate or Isothiocyanates and Schizophrenia

Several clinical studies examined the efficacy of GLS and ITCs, particularly GRA and SFN, in treating cognitive deficits and symptoms of schizophrenia.

A pilot study evaluated the effect of daily supplementation with 30 mg of GRA for eight weeks in schizophrenic patients treated with antipsychotics. The study found a significant improvement in the Accuracy component of the One Card Learning Task (OCLT), a test assessing visual recognition, attention, and short-term memory. Although no significant changes were observed in other cognitive parameters measured using the CogState battery or in scores on the Positive and Negative Syndrome Scale (PANSS), and no variations were noted in BDNF levels, the results suggest a potential beneficial effect on cognitive function, with no relevant adverse effects [176]. In a subsequent study, a randomized, double-blind, placebo-controlled trial (NCT02810964) involved 64 participants, 58 of whom completed the protocol. They were treated for 16 weeks with broccoli extracts containing GRA + MYR. The GRA + MYR-based extracts did not result in significant differences between the treated and placebo groups in either psychotic symptoms or cognitive functions, as measured by the PANSS and the MATRICS Consensus Cognitive Battery (MCCB). However, the authors suggested that future studies should explore higher doses of SFN and include patients at an earlier stage of the disease to better determine treatment efficacy [177]. Another 22-week randomized, double-blind study found that administration of GRA and MYR led to significant improvements in three secondary outcome cognitive domains: spatial working memory, verbal learning, and reasoning/problem-solving, although it did not show significant effects on the MCCB global composite score (primary outcome). These improvements ranged from small to moderate in magnitude and varied depending on the dosage, suggesting a direct effect of SFN on cognitive function, rather than an indirect improvement linked to psychotic symptom control. Additionally, in the higher-dose GRA group, a slight reduction in Calgary Depression Scale scores was observed, with a more pronounced effect in female patients [178]. Finally, another study (NCT03451734) examined the efficacy of SFN in patients with predominant negative symptoms, using a dose of 90 mg/day for 24 weeks in addition to standard antipsychotic therapy. The results showed a significant reduction in the PANSS negative subscale total score, as well as improvements in SOD activity and high-sensitivity C-reactive protein (HsCRP) levels, suggesting that SFN may exert an anti-inflammatory effect, which could be beneficial in treating negative symptoms. However, no significant effects were found on positive symptoms of PANSS [179]. Overall, these studies suggest that while GLS and ITCs have not shown significant direct effects on global psychotic symptoms, they may have a positive impact on certain cognitive functions and negative symptoms of schizophrenia. Therefore, while the results are promising, further studies are needed to confirm their potential as adjunctive treatments for cognitive deficits and negative symptoms, as well as to optimize dosages and administration methods. An overview of the described studies is presented in Table 8. 

### 6.3. Depression and Anxiety

Depression and anxiety are among the most prevalent psychiatric disorders worldwide and represent a leading cause of disability and reduced quality of life [180,181]. Depression is characterized by persistent sadness, loss of interest in daily activities, changes in appetite and sleep patterns, and suicidal thoughts. Unfortunately, available antidepressant treatments are not always effective, and many patients experience relapses or undesirable side effects [182]. According to the World Health Organization, more than 350 million people suffer from depression, with a significant impact on public health [183,184]. Although the precise pathophysiological mechanisms of depression are not yet fully understood, numerous findings suggest a key role of inflammation in its onset and progression [185]. At the same time, chronic stress is known to contribute to the development of depressive and anxiety disorders through the hyperactivation of the hypothalamic–pituitary–adrenal (HPA) axis, leading to an increased release of corticosteroids and dysregulation of the immune and inflammatory response [186]. Currently, pharmacological treatments for depression primarily include selective serotonin reuptake inhibitors (SSRIs), which are considered first-line treatment for depressive symptoms due to their good tolerability [187]. However, despite their widespread use, several challenges remain in their application, including non-response in a significant proportion of patients and the presence of intolerable side effects, such as sexual dysfunction, sleep disturbances, and, in some cases, undesirable cardiovascular effects [188,189]. Furthermore, SSRI treatment requires a 4–6 weeks period before clinical improvements become apparent [190]. These limitations have driven the exploration of new pharmacological strategies to achieve faster symptom relief. In recent years, research has focused on the role of nutrition in the prevention and treatment of depression and anxiety. High intake of fruits, vegetables, fish, and whole grains has been associated with a reduced risk of developing depressive disorders [191]. In this context, bioactive natural compounds, such as GCLs and ITCs, have gained increasing attention for their potential neuroprotective and anti-inflammatory effects. Specifically, ITCs, particularly SFN and GRA, showed promising effects in the treatment of depression and anxiety through the modulation of various neurobiological mechanisms.

#### 6.3.1. Preclinical Studies on Glucosinolate or Isothiocyanates and Depression/Anxiety

Depression is often associated with reduced neuronal plasticity, particularly in brain regions involved in mood and emotion regulation, such as the hippocampus and prefrontal cortex. An in vitro study showed that SFN enhanced neuronal plasticity by promoting neurite outgrowth in PC12 cells in a concentration-dependent manner. The inhibition of Nrf2 via siRNA significantly attenuated this effect, confirming the crucial role of Nrf2 in this process. SFN also increased Nrf2 protein levels, and this increase was blocked by Nrf2 siRNA, further supporting its involvement in neurite growth [192]. In a mouse model of acute and chronic stress, repeated SFN administration, particularly at a dose of 10 mg/kg/day, exerted antidepressant and anxiolytic effects. In behavioral tests, repeated SFN treatment for 14 days significantly reduced immobility time in the tail suspension test (TST) at 10 mg/kg/day and the forced swim test (FST) at 3 mg/kg/day and 10 mg/kg/day, improving depressive symptoms in mice subjected to chronic mild stress, with antidepressant effects comparable to fluoxetine. Furthermore, SFN at 10 mg/kg/day increased sucrose preference in the sucrose preference test (SPT), indicating an improvement in anhedonia, and reduced anxiety-like behaviors in the open field test (OFT) at 10 mg/kg/day while decreasing latency to feed in the novelty-suppressed feeding test (NSF) at 3 mg/kg/day and 10 mg/kg/day. At the neurobiological level, SFN regulated the HPA axis, lowering serum corticosterone and adrenocorticotropic hormone (ACTH) levels. It also counteracted stress-induced increases in pro-inflammatory cytokines such as IL-6 and TNF-α, demonstrating anti-inflammatory action [146]. In a social defeat stress model, SFN pre-treatment mitigated reductions in social interaction and sucrose preference, indicating protective effects against social avoidance and anhedonia. These improvements were associated with Nrf2 pathway activation, restoration of BDNF levels, and modulation of its receptor TrkB signaling in key brain regions such as CA3, dentate gyrus (DG), and prefrontal cortex (PFC), suggesting enhanced neuronal function [192]. Moreover, GRA treatment in the juvenile and adolescent phases prevented depressive behaviors in adulthood [192]. In a mouse model of LPS-induced depression, preventive treatment with SFN significantly reduced TNF-α and increased IL-10, demonstrating anti-inflammatory effects. Behaviorally, SFN dose-dependently reduced immobility time in the TST and FST on LPS-treated mice. At the molecular level, SFN counteracted alterations in BDNF, PSD-95, and GluA1 levels, key proteins for neuroplasticity, restoring their levels in the PFC and hippocampus, thereby improving neuroplasticity and preventing LPS-induced dendritic spine loss in these regions, a phenomenon associated with depression [193]. Similarly, GRA supplementation (0.1%) during youth prevented the onset of depressive behaviors induced by LPS in adulthood, suggesting preventive LPS-induced depressive behaviors in adulthood by preserving BDNF and synaptic protein levels, while also preventing excessive spine density increases in the nucleus accumbens (NAc). Furthermore, GRA supplementation (0.1%) between the 5th and 8th week of life significantly attenuated the increase in immobility time in the TST and FST in adulthood without affecting control mice behavior [193]. SFN has also shown analgetic and antidepressant effects in chronic neuropathic pain models, reducing nociceptive responses, mechanical and thermal hypersensitivity, and depression-like behaviors. These effects were mediated by Nrf2 activation, increased HO-1 expression, and reduced oxidative stress, along with the modulation of MAPK proteins (JNK, ERK1/2, p-38), which are involved in inflammatory and neuroplasticity processes. Furthermore, SFN enhanced morphine’s analgesic efficacy by counteracting neuropathy-induced reductions in opioid receptors [194].

In an in vivo Alzheimer’s disease-associated depression model, SFN counteracted depression-like effects induced by β-amyloid oligomers (AβOs), improving performance in the FST, OFT, and SPT of rats. This effect was accompanied by reduced oxidative stress (decreased MDA levels), neuroinflammation (reduced IL-1β and TNF-α levels), and preservation of the serotonergic system (increased TPH2 and SERT levels), suggesting a neuroprotective effect [96]. Similarly, GRA exhibited significant antidepressant properties in a depressive phenotype rat model, restoring serotonin and norepinephrine levels while reducing NF-κB activation, kynurenine production, and ROS levels in the PFC [195]. The daily intraperitoneal administration of slow-releasing hydrogen sulfide donors, such as AITC and phenyl isothiocyanate (PITC), has demonstrated positive effects on depression associated with chronic osteoarthritis pain, induced by intra-articular injection of monosodium iodoacetate, in female C57BL/6 mice. Specifically, both treatments significantly reduced the immobility time in behavioral tests used to assess depressive symptoms in osteoarthritis mice, showing a clear antidepressant effect. These effects were also associated with modulation of antioxidant enzyme levels (HO-1, NQO1, GSTM1, GSTA1). However, in tests specifically designed to assess anxiety (EPM and OFT), neither AITC nor PITC were able to normalize the anxiety-like behaviors induced by osteoarthritis [196]. Subsequently, the same research group demonstrated that AITC and PITC were able to significantly reduce depressive-like behaviors also associated with chronic neuropathic pain induced by chronic constriction of the sciatic nerve in male C57BL/6 mice. Specifically, in the TST, mice with neuropathic pain exhibited increased immobility time, indicative of depressive behavior. Interestingly, administration of AITC and PITC reduced this parameter in both the nerve-injured mice and the control group, suggesting an antidepressant effect that is independent of the presence of chronic pain [197]. These findings suggested that slow-releasing hydrogen sulfide donors, despite not being effective in alleviating anxiety related to chronic pain, may represent a promising strategy for the treatment of depression associated with osteoarthritis and neuropathic pain. SFN’s rapid antidepressant effects were further confirmed in murine models, where it attenuated LPS-induced reductions in Nrf2 and BDNF levels, counteracting the increased expression of BDNF transcriptional repressors (HDAC2, mSin3A, and MeCP2) in the mPFC and hippocampus. These effects correlated with improved synaptic transmission and reduced depressive behaviors, but were absent in Nrf2 knockout mice, confirming the essential role of this pathway [198]. In the chronic social defeat stress (CSDS) model, SFN promoted stress resilience by regulating Nrf2 and MeCP2 in the mPFC and microglia, leading to increased BDNF expression and dendritic spine restoration. Additionally, SFN reduced pro-inflammatory microglia (iNOS^+^) while activating the anti-inflammatory phenotype (arginase1^+^), leading to behavioral improvements in the social interaction test (SIT), FST, and SPT, reinforcing its therapeutic potential for depression [199]. Further studies suggested that Nrf2 activation via SFN restored TREM2 expression, enhanced the anti-inflammatory microglial phenotype (arginase 1^+^), and improved dendritic spine density in the mPFC of CSDS-exposed mice, highlighting a possible therapeutic mechanism for depression. The Nrf2-TREM2 pathway appeared crucial in BDNF-TrkB signaling modulation, essential for synaptic plasticity and stress resilience [200]. Beyond SFN, an aqueous extract of Raphanus sativus sprouts (AERSS), rich in ITCs (sulforaphene, SFN, and iberin), demonstrated anxiolytic effects in experimental models. Treatment with 30 mg/kg and 100 mg/kg i.p., or 500 mg/kg orally (p.o.), significantly increased time spent in the open arms of the elevated maze, comparable to diazepam and buspirone. Mechanistic studies suggested that these effects involve GABAA/BDZs and 5-HT1A receptors, implicating both the GABAergic and serotonergic systems [201]. In a mouse model of olfactory bulbectomy (OB)-induced depression, SFN (10 mg/kg for 14 days) significantly improved depressive behaviors, reducing hyperactivity and normalizing self-care and motivation. Biochemical analysis revealed that SFN enhanced total antioxidant capacity (TAC) in the frontal cortex and serum, increasing SOD activity [202]. Collectively, these preclinical studies suggest that GLS and their derivates, like SFN, offer a promising strategy for the prevention and/or treatment of depression and anxiety. Their beneficial effects appear to stem from a combination of anti-inflammatory, antioxidant, and neurotrophic properties, with the ability to modulate the HPA axis, serotonergic and noradrenergic neurotransmission, BDNF signaling, and synaptic plasticity. Additionally, dietary GRA supplementation could also offer a long-term preventive approach, reducing mood disorder vulnerability. An overview of the described studies is presented in Table 9.

#### 6.3.2. Clinical Study on Sulforaphane in Depression/Anxiety 

There are multiple physical and mental factors underlying the development of depressive symptoms, and depression is a particular concern among patients undergoing major surgical procedures. In a double-blind, randomized, placebo-controlled trial, the efficacy and safety of SFN in the treatment of depression induced by cardiac surgery, such as percutaneous coronary intervention (PCI) and coronary artery bypass grafting (CABG), was evaluated. After six weeks of treatment, patients (*n* = 30) treated with a tablet containing 30 mg/day of SFN (appro. 169.2 μmoL/day of SFN) showed a significant improvement in Hamilton Rating Scale for Depression (HAM-D) scores compared to the placebo group (*n* = 30). The remission rate was also higher in the SFN group, although the difference was not statistically significant. Additionally, SFN treatment was well tolerated, with no significant differences in common side effects between the groups. These findings suggest that SFN could be a safe and effective therapy for alleviating depressive symptoms in patients undergoing cardiac surgery. However, further studies with larger samples and longer follow-up periods are needed to confirm these effects [203].

### 6.4. Epilepsy

Epilepsy is a chronic neurological disorder characterized by spontaneous and recurrent seizures, often associated with abnormal, hypersynchronous neuronal discharges in the brain [204]. While epilepsy can arise from diverse etiologies, temporal lobe epilepsy (TLE) is among the most common and clinically significant forms, particularly due to its frequent association with cognitive dysfunctions that severely impact patients’ quality of life [205]. Approximately 30% of epilepsy cases are classified as drug-resistant, underscoring the urgent need for more effective and targeted therapies [206]. This need is particularly acute in pediatric populations, where seizures may disrupt brain maturation and result in long-term neurodevelopmental impairments [207]. In recent years, increasing attention has been paid to the role of oxidative stress and mitochondrial dysfunction in the pathophysiology of epilepsy, both in adult and immature brains [208]. Persistent oxidative damage can contribute to neuronal injury, synaptic dysfunction, and cognitive deficits observed in both experimental and clinical epilepsy [209]. Accordingly, antioxidants have emerged as potential neuroprotective agents, especially those capable of activating endogenous defense mechanisms, such as the Nrf2/HO-1 signaling pathway [210]. Natural compounds derived from dietary sources, such as GLS derivatives and ITCs have shown promise in modulating oxidative and inflammatory pathways, making them attractive candidates for complementary therapeutic strategies [210].

#### Preclinical Studies on Glucosinolate or Isothiocyanates in Epilepsy

Activation of the Nrf2–ARE signaling pathway by SFN has demonstrated notable neuroprotective effects in various epilepsy models. In a chronic amygdala kindling model, SFN treatment suppressed seizure progression, reduced post-discharge duration, and significantly improved seizure-induced cognitive deficits, as assessed by the Morris water maze test. These benefits correlated with decreased oxidative stress markers, lower MDA levels, and higher hippocampal GSH content, as well as the upregulation of Nrf2 nuclear translocation alongside increased expression of antioxidant enzymes HO-1 and NQO1. The absence of such effects in Nrf2-deficient mice underscores the pivotal role of Nrf2 in mediating SFN’s neuroprotection [211]. Further studies confirm SFN’s anticonvulsant and antioxidant properties across multiple seizure models, where pre-treatment raised seizure thresholds and reduced status epilepticus (SE) incidence. These effects were linked to enhanced systemic and hippocampal antioxidant defenses, including elevated SOD activity and improved mitochondrial respiration, specifically boosting ATP-linked oxygen consumption and restoring mitochondrial complexes I and II activity SE-impaired. However, SFN did not prevent hippocampal neuronal loss post-SE, suggesting that early bioenergetic improvements may not fully confer neuroprotection in severe epilepsy models [212]. Despite its generally favorable safety profile, high doses of SFN (200 mg/kg) exhibited proconvulsant effects by lowering seizure thresholds and potentiating carbamazepine’s anticonvulsant efficacy through possible pharmacokinetic interactions. Toxicity signs included sedation, hypothermia, neuromuscular impairment, mortality (LD_50_ of 212.67 mg/kg), and leukopenia, highlighting the importance of dose considerations and safety evaluations for clinical translation [213]. Combinatorial antioxidant therapy with SFN and NAC during epileptogenesis in a rat SE model delayed seizure onset, prevented disease progression, and rescued cognitive function. This treatment normalized hippocampal redox balance, prevented mitochondrial dysfunction, reduced oxidative and inflammatory damage (including HMGB1 translocation), and attenuated neuronal loss, outperforming either agent alone. These results indicate that early antioxidant intervention may offer lasting disease-modifying effects [214]. In vitro and in vivo temporal lobe epilepsy models further support SFN’s neuroprotective and antioxidant effects via Nrf2 activation, with restored GSH levels, reduced ROS production, increased antioxidant capacity, and diminished hippocampal neuronal loss [215]. Similarly, in immature rats subjected to Li-pilocarpine SE, SFN improved brain metabolism and cerebral blood flow without altering seizure severity or acute neuronal death, suggesting its benefits derive from enhancing metabolic and vascular responses during epileptogenesis [216]. In another lithium–pilocarpine SE model in immature rats, SFN pre-treatment (two doses of 5 mg/kg, 48 h and 24 h before SE) significantly reduced oxidative stress and mitochondrial dysfunction. SFN attenuated superoxide production and levels of 3-nitrotyrosine and 4-hydroxynonenal during both acute and post-SE periods (up to 3 weeks) and partially preserved mitochondrial complex I activity. These effects occurred without modifying seizure severity, indicating that SFN’s protective actions may rely more on Nrf2-mediated mitochondrial support than on direct anticonvulsant activity [217]. Other ITCs, such as SIN, demonstrated anticonvulsant, antioxidant, and anti-inflammatory effects in pentylenetetrazole-treated rats by delaying seizure onset, improving memory, increasing antioxidant enzyme levels (SOD and CAT), and downregulating pro-inflammatory genes (Il1b and Nlrp3) [218]. Moreover, BITC treatment in a lithium–pilocarpine epilepsy model improved cognitive and motor functions, reduced neuronal loss, and enhanced Nrf2 pathway activity and antioxidant defenses, suggesting its therapeutic potential against epilepsy-related neurodegeneration [219]. Overall, preclinical evidence indicates that GLS and ITCs exert multifaceted neuroprotective effects in epilepsy models primarily via Nrf2-mediated antioxidant and metabolic modulation. While these compounds show promise as adjunctive therapies to mitigate oxidative stress and cognitive decline in epilepsy, further research is necessary to clarify optimal dosing and long-term safety. An overview of the described studies is presented in Table 10.

## 7. Adverse Effects

GLS and ITCs generally present a favorable safety profile, but their consumption at high doses can lead to adverse effects. Preclinical studies showed that high amounts of GRA can suppress phase I enzymes in the liver and lungs, affecting the metabolism of endogenous substances and increasing the risk of bioactivation of polycyclic aromatic hydrocarbons (PAHs) into carcinogenic forms [220,221]. Moreover, a GLS-rich diet led to reduced food intake and growth in rats, with increased mortality and alterations in thyroid function, including thyroid gland enlargement and decreased plasma levels of thyroid hormones [222]. However, moderate consumption of Brassicaceae vegetables does not appear to pose a significant clinical risk, especially in individuals with adequate iodine intake [223]. Regarding SFN, some clinical studies have reported gastrointestinal disturbances and a burning sensation in the throat following ingestion at high doses, effects that can be mitigated with gradual exposure [224]. The adverse events reported were generally mild, such as nausea or vomiting associated with the odor or ingestion of SFN preparations [35]. Moreover, ITCs showed a dose-dependent effect on the generation of ROS and the breakage of double-stranded DNA, contributing to cytotoxic effects in cancer cells, but being well tolerated in normal cells due to more efficient DNA repair mechanisms [225,226,227,228,229]. Even in studies with patients suffering from neuropsychiatric disorders, treatment with SFN-rich extracts was well tolerated, with no significant differences in adverse events between treated and placebo groups, despite some serious adverse events being reported [177]. Additionally, a clinical trial showed a high completion rate (73.77%), as well as no evidence of significant adverse events related to the administration of SFN, as confirmed by routine tests on liver, kidney, and metabolic function [179]. In conclusion, GLS and ITCs are generally well tolerated and offer numerous health benefits, thanks to their antioxidant, anti-inflammatory, and potentially chemopreventive properties. Although excessive consumption may require caution, particularly in individuals predisposed to thyroid dysfunction or those exposed to environmental mutagens, clinical studies suggest that moderate doses are safe and free from significant adverse effects. With balanced use, these compounds could represent valuable nutritional support for the prevention and management of various pathological conditions, with a generally favorable safety profile.

## 8. Clinical Challenges and Future Perspectives

Despite the growing body of evidence supported by numerous preclinical studies and emerging clinical data, the clinical translation of ITCs remains limited and requires further investigation. One of the main challenges is the definition of effective and safe dosage for humans. In clinical studies, widely variable doses of ITC have been used; for example, SFN has been administered in doses ranging from below 25 to above 500 μmol/day, commonly delivered through broccoli sprout extracts or standardized supplements. However, inter-individual differences in metabolism influenced by the activity of gut microbiota and glutathione-S-transferase enzyme activity can significantly affect ITCs’ bioavailability and therapeutic response. Moreover, administration methods play a crucial role in therapeutic outcomes. Oral administration is currently the most studied and practical route, but it is susceptible to variability due to food matrix effects, individual absorption, as well as dietary and metabolic factors. Promising strategies include the use of standardized extracts with known GLS-to-ITC conversion rates, or encapsulated formulations to improve stability and bioavailability. Patient selection also plays a critical role. Individuals with elevated levels of oxidative stress or systemic inflammation, common features in many neurodegenerative and psychiatric disorders, are likely to benefit the most from ITC-based interventions. However, this remains a hypothesis that requires validation through clinical trials designed with stratified patient cohorts and guided by specific biomarkers in order to identify those subpopulations that respond most effectively to ITC supplementation. To enable successful clinical translation, future trials should focus on defining therapeutic windows, optimizing formulations, and identifying predictive biomarkers of response to ensure effective clinical use. Moreover, long-term safety studies are essential to evaluate potential adverse effects and drug–nutrient interactions, especially in combination with conventional pharmacological treatments. Mechanistic research should also be extended to lesser-known ITCs to better characterize their molecular targets, particularly in relation to brain-specific signaling pathways and epigenetic modulation. Integrating multi-omics approaches and personalized medicine frameworks may further improve our understanding of individual variability in ITC response and support more tailored neuroprotective strategies.

## 9. Methods

All articles included in this review were selected through the PubMed databases, considering publications from 2014 to 2024. The research was conducted using the following keywords: “isothiocyanate” or “glucosinolate” or “sulforaphane” or “glucoraphanin” or “moringin” in combination with “neuroinflammation” or “alzheimer” or “parkinson” or “multiple sclerosis” or “autism” or “schizophrenia” or “depression” or “anxiety” or “epilepsy” (and other related terms). From these search criteria, a total of 412 articles were found, 84 of which were excluded as they were reviews, resulting in a total of 328 studies that were examined by two reviewers. The inclusion criteria focused exclusively on original studies, written in the English language, investigating naturally occurring plant-derived ITCs and GLS and their effects on neuropsychiatric disorders, neurodegenerative diseases, neurodevelopment diseases, and other brain health-related conditions. All articles related to the use of ITCs in experimental assays, such as fluorescein isothiocyanate (FITC) or Rhodamine B isothiocyanate (RITC), as well as those centered on other non-relevant phytochemicals, were excluded. At the end of the selection process, 142 articles were selected, of which 128 preclinical studies and 15 clinical studies were collected.

## 10. Conclusions

**Figure 2:**
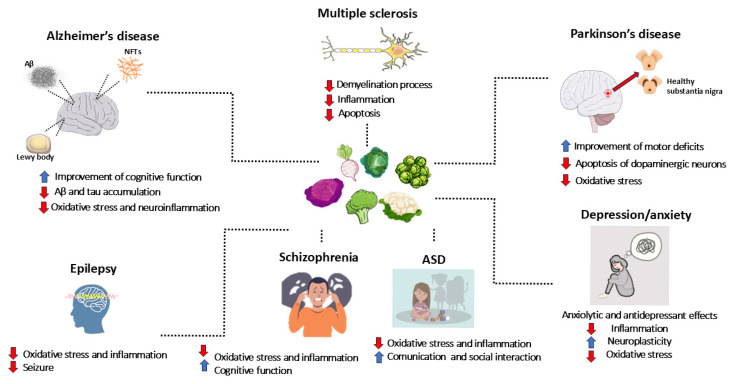
Potential benefits of glucosinolate derivatives in neurodegenerative diseases, neurodevelopmental disorders, and psychiatric conditions: insights from preclinical and clinical studies. The figure was created using the vector image bank of Servier Medical Art by Servier (http://smart.servier.com/ (accessed on 1 April 2025)). Servier Medical Art is licensed under CC BY 4.0.

**Figure 3:**
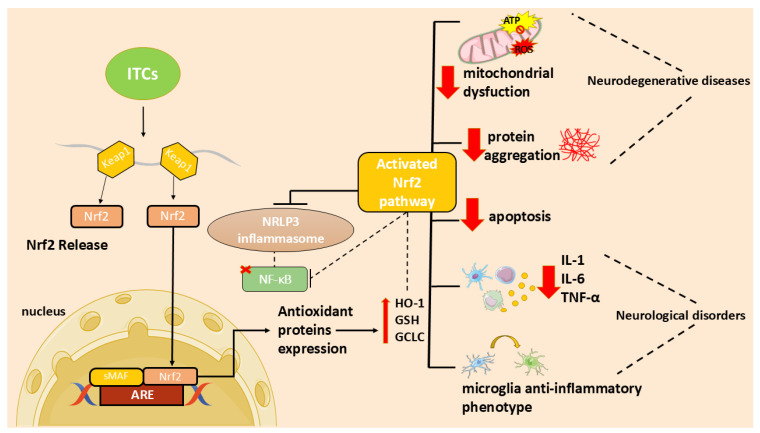
Overview of ITC effects on the Nrf2 pathway and their anti-inflammatory actions. Both antioxidant and anti-inflammatory properties mediate their neuroprotective actions. However, in neurodegenerative diseases, the dominant mechanism of ITCs involves antioxidant actions, while in neurological disorders the immunoregulatory effect is prevalent.

This review provides an in-depth description of preclinical and clinical studies conducted over the past ten years on the effects of GLS and ITCs in neurological, neurodevelopmental, and psychiatric disorders as well as epilepsy (Figure 2). The molecular mechanisms underlying their beneficial effects and their potential positive impact on patients’ quality of life were highlighted. The collected evidence suggests that ITCs are bioactive molecules of great interest for the prevention and treatment of numerous neurodegenerative, neurodevelopmental, and psychiatric disorders. Preclinical research and early clinical studies indicate that these compounds may exert neuroprotective effects through antioxidant mechanisms (primarily via activation of the Nrf2 pathway) and anti-inflammatory actions, helping to mitigate conditions such as depression, schizophrenia, ASD, AD, PD, and MS (Figure 3). For these reasons they may represent an alternative pharmacological strategy also in association with standard therapies. Although each disease discussed in this review has distinct clinical and pathological features, it is evident that ITCs exert beneficial effects primarily through two core mechanisms: modulation of oxidative stress and immune regulation. In neurodegenerative diseases, such as AD, PD, MS, and epilepsy, the dominant mechanism of ITCs involves the activation of the Nrf2 pathway. This pathway enhances cellular antioxidant defenses, promotes mitochondrial integrity, and supports proteostasis, all of which are crucial in counteracting protein aggregation and neuronal degeneration. In contrast, in psychiatric and neurodevelopmental disorders, such as schizophrenia, depression, and ASD, ITCs appear to act more prominently on immune regulation. These effects include the suppression of pro-inflammatory cytokines, inhibition of NF-κB activation, and promotion of anti-inflammatory phenotypes in microglia. Interestingly, some mechanisms overlap. For example, Nrf2 activation also indirectly regulates inflammation, while modulation of immune responses can reduce oxidative stress. This dual action suggests that ITCs may target shared pathophysiological mechanisms across these disorders, although with different predominant effects depending on the type of disease. A comparative analysis thus reveals that while antioxidant pathways (e.g., Nrf2, HO-1 induction) are central in neurodegenerative disorders and epilepsy, immune-modulatory effects (e.g., inhibition of NF-κB, cytokine regulation) are more pronounced under psychiatric and neurodevelopmental conditions. Understanding these nuanced differences will be essential for tailoring therapeutic strategies based on disease-specific pathological hallmarks.

While preclinical data from animal and in vitro studies are promising, further clinical research with large-scale trials is needed to determine the true therapeutic potential of these compounds and establish their optimal administration in humans, particularly for diseases such as AD, PD, MS, and epilepsy, where clinical evidence remains limited. Of note, SFN is the most studied ITC, and then the molecular mechanisms underlying its neuroprotective actions were discovered. Instead, more studies are needed for less-indagated ITCs to deepen the knowledge on their protective actions. In the meantime, integrating cruciferous vegetables into the diet may represent a safe and potentially effective nutritional strategy to enhance brain health and reduce the risk of neurological and psychiatric disorders. Moreover, the consumption of cruciferous vegetables provides GLS and other beneficial phytocompounds, including flavonoids and phenols, which may synergistically enhance neuroprotective effects.

## Figures and Tables

**Table 1:** Preclinical studies: results on the effects of glucosinolate or isothiocyanate treatments in neuroinflammation and oxidative stress models.

Compounds	In Vitro Model	Dose	Treatment Duration	Main Findings	Ref.
**SFN**	BV2 cells treated with LPS (100 ng/mL) for 6 h and 24 h	5–10 μM	1, 3, 6, 12, 24 h	Inhibition of nitrite production, iNOS, and COX-2 expression; modulation of the MAPK pathway.	[39]
**SFN**	BV-2 cells treated with LPS (0.5 mg/L) for 12 h	5, 10, and 15 μM	12 h	Inhibition of NF-kB and the release of inflammatory mediators. Inhibition of necroptosis mediated by JNK E p65.	[40]
**SFN**	N9 microglia cells treated with LPS (1 μg/mL) for 4 h	5 μM	1 h before LPS	Inhibition of NLRP3 inflammasome.	[41]
**SFN**	BV-2 cells treated with MGO-AGEs (1 mg/mL)	1–20 μM	24 h	Reduction in ROS. Inhibition of NF-κB activation and the release of pro-inflammatory cytokines.	[42]
**SFN**	Primary microglia isolated from young and adult Balb/c mouse and BV-2 microglia cell line treated with LPS (100 ng/mL) for 8 h	2.5 μM	Primary microglia: pre-treatment for 1 h before LPS BV-2 cell line: 1, 6, 9, 24 h	Upregulation of ARE genes including NQO1, HMOX1, and GCLM in both cell lines. Reduction in pro-inflammatory markers in primary microglia from adult and aged mice.	[43]
**SFN**	SH-SY5Y cells treated with CPF (100 μM) for 24 h BV2 cells treated with LPS (500 ng/mL) for 24 h	5 μM	6–24 h	Downregulation of IL-1β, TNF-α, and PGE2. Increased HO-1 expression.	[44]
**SFN**	Primary microglia treated with LPS (1 μg/mL) for 24 h	5, 10, and 30 μM	1, 3, and 5 h	Induction of reversible elongations of microglia; increased expres-sion of Akt.	[45]
**SFN**	BV2 cells treated with LPS (1 μg/mL) for 24 h	20 μM	24 h	Inhibition of NF-κB pathway.	[46]
**SFN**	Astrocytoma cells transfected with Nrf2 short interference RNA (siRNA) and treated with OKA (20 nM) for 24 h	10 μM	24 h	Increased cell viability; restored Nrf2, HO-1, and GCLC expression.	[48]
**SFN** **NAC**	BV2 cells and primary cortical neuronal culture treated with LPS (200 ng/mL) + IFN-γ (20 ng/mL) for 48 h	SFN: NAC concentrations: 1 μM:10 μM or 5 μM:50 μM	48 h	Increased viability; reduction in nitrite level and of TNF-α.	[50]
**SFN**	BV2 cells and primary microglia of aged mouse treated with LPS (100 ng/mL)	2.5 μM	3, 6, 9, and 24 h	Nrf2 activation and reduction in IL-6 and IL-1β.	[51]
**SFN-Enriched Broccoli Sprouts**	BV-2 cells treated with LPS (100 ng/mL) for 24 h	100 μg/mL	24 h	Inhibition of NF-κB and of the secretion of pro-inflammatory pro-teins (iNOS, COX-2, TNF- α, IL-6, IL-1β, PGE2). Increased expression of Nrf2 and HO-1 and reduction in apoptosis.	[55]
**BITC**	BV2 cell treated with LPS (1 μg/mL) for 3 h and 48 h	1, 5 and 10 μM	1 h before LPS	Reduced IL-1β protein levels. Inhibition of NLRP3 and IL-1β.	[57]
**ESE**	NSC-34 motor neurons treated with the medium of LPS-stimulated RAW 264.7 (1 μg/mL) for 24 h	0.1, 0.2, 0.3 e 0.4 μg/mL	24 h pre-treatment	Increased expression of IL-10. Reduction in apoptosis and TNF-α expression.	[58]
**GMG-ITC**	SH-SY5Y treated with H_2_O_2_ (300 μM) for 4 h	1.25 μg/mL	72 h	Reduction in ROS through Nrf2 activation; modulation of the MAPK pathway and reduction in NF-κB levels.	[59]
**AITC**	BV2 cells treated with LPS (100 ng/mL) for 24 h	1, 5, 10, 20 μM	30 min before LPS	Inhibition of COX-2, iNOS, TNF-α, IL-6, PGE2, and NO.	[60]
**AITC,** **PEITC, SFN**	Astrocytes from the neocortical tissues of 1-day-old Wistar rats treated with LPS (10 μg/mL) for 20 h	AITC: 5, 25, 50, 400 μM PEITC and SFN: 5, 25, 50 μM	20 h	Inhibition of MMP1 and MMP3 release through modulation of the ERK pathway.	[61]
**ITH12674**	Cortical neuron treated with TBH (30 μM) for 24 h	0.1, 0.3, and 1 μM	24 h	Reduction in ROS. Increased GSH levels.	[62]
**ITH12674**	Primary glial cell treated with LPS (1 μg/mL) for 18 h	10 μM	18 h	Reduction in IL-1β, TNFα, and NF-κB. Increased expression of Nrf2 and HO-1.	[63]
**Compounds**	**In Vivo Model**	**Dose**	**Treatment Duration**	**Main Findings**	**Ref.**
**SFN**	C57BL6/J mice treated with LPS (100 μg/kg) for 5 days	5 mg/kg	5 days	Increased expression of Akt and elongation in microglia cells.	[45]
**SFN**	C57BL/6 mice treated with LPS (0.25 mg/kg) for 7 days	20 mg/kg	7 days	Improvement in learning and memory deficits. Modulation of the mTOR pathway, increasing BDNF expression.	[47]
**SFN**	Male Sprague–Dawley rats OKA-injected (200 ng) for 2 days	5 mg/kg	1 h prior to and 24 h after OKA administration	Improvement in memory function.	[48]
**SFN**	B6.129P-Cx3cr1tm1Litt/J) mice; Cx3cr1+/+ mutant for hTAUP301L	50 mg/kg	3 weeks	Inverted astrogliosis.	[49]
**SFN** **NAC**	Male Sprague–Dawley rats with induction of lateral FPI	NAC: 500 mg/kg; SFN: 5 mg/kg	13 days	Reduction in pro-inflammatory biomarkers.	[50]
**SFN**	Balb/c mice treated with LPS (1 μg)	50 mg/kg	3 days	Reduction in pro-inflammatory mediators in hippocampus and liver.	[51]
**SFN**	C57BL/6J wild type; Pde6brd10 mice	20 mg/kg	7 days	Reduction in glial cell activation. Reduction in IL-1β and retinal degeneration.	[52]
**SFN**	Male Wistar rats treated with ammonium diet for 6 weeks	0.5 mg/kg	6 weeks	Reduction in astrocyte and microglia activation. Normalized extracellular GABA. Reduction in pro-inflammatory cytokines.	[53]
**SFN**	Male C57Bl/6N mice tMCAO model	25 mg/kg body weight	3, 7 and 23 h after tMCAO	Inhibition of inflammasomes causes anti-inflammatory effects in ischemic stroke.	[54]
**Broccoli sprouts enriched with SFN**	Male ICR mice treated with SCOP (1.2 mg/kg)	200 mg/kg	2 weeks	Nrf2 activation. Inhibition of caspase-3 and reduction in neuronal apoptotic process.	[55]
**10% Broccoli diet**	Adult (4-month-old) and aged (18-month-old) BALB/c mice treated with LPS (0.33 mg/kg) for 24 h	AIN-93M; AIN-93M + 10% freeze-dried broccoli	24 days	Reduction in astrocyte markers and oxidative stress.	[56]
**ITH12674**	Sprague–Dawley rats and C57/BL6 j mice treated with LPS (0.5 mg/kg)	1 mg/kg	2 h before LPS	Improvement in locomotion and social interaction. Reduced expression of IL-1β, TNF-α, GFAP, IBA1, and CD68.	[63]

Chlorpyrifos (CPF); glucomoringin-isothiocyanate (GMG-ITC); methylglyoxal-advanced glycation and products (MGO-AGEs); N-acetylcysteine (NAC); okadaic acid-induced model (OKA); temporary middle cerebral artery occlusion (tMCAO); scopolamine (SCOP); LPS; IFN-gamma; tert-butyl hydroperoxide (TBH); lateral fluid-percussion model (FLP); sulforaphane (SFN); NAC; allylisothiocyanate (AITC); phenylethyl-isothiocyanate (PEITC), Eruca sativa seed extract (ESE); benzylisothiocyanate (BITC).

**Table 2:** Preclinical studies: results on the effects of glucosinolates or isothiocyanates treatments in AD models.

Compounds	Computational and Investigational Studies		Main Findings		Ref.
**RB and PB**	Docking study		Anti-cholinesterase activities (AChE and BuChE inhibitory activities).		[70]
**SFN**	In silico study using different database		Identification of 45 targets involved in the pathogenesis of AD.		[69]
**SFN**	LC/ESI-MS		Less inclination of Aβ to aggregate.		[71]
**SFN**	In silico docking simulation		BACE1 inhibitor.		[72]
**Compounds**	**In Vitro Model**	**Dose**	**Treatment Duration**	**Main Findings**	**Ref.**
**SFN**	HEK293T transfected with NFE2L2 and HT22 cells	15 μM	12 h	Modulation of autophagy.	[73]
**SFN**	CN1.4 cells transfected by GFP and GFP-tau (s) form	10 μM	24 h	Reduction in ROS. Increased antioxidant gene expression.	[74]
**SFN**	N2a/APPswe cells	1.25 and 2.5 mΜ	48 h	Epigenetic modification.	[75]
**SFN**	PC12 cells treated with SNP (350–400 μM)	1 μM	24 h	Increased cell viability in the model of nitrosative stress.	[76]
**SFN**	EOC-20 microglial cell line treated with Aβ (100 ng/mL, 500 ng/mL, and 1000 ng/mL) for 24 h	5 µM	24 h	Increased phagocytic activity.	[77]
**SFN**	Primary cortical neuronal cells derived from 3 × Tg-AD mouse	10 µM	6 h	Increased CHIP levels and decreased Aβ accumulation levels.	[78]
**Broccoli Sprouts Juices**	SH-SY5Y treated with Aβ_25–35_ (25 μM) for 24, 48, 72 h	10 μL/mL	24, 48, 72 h	Inhibition of apoptosis.	[79]
**SFN**	Astrocytes from neonatal C57BL/6J mouse	0, 0.1, 0.25, 0.5, 2.5, 5, and 10 μM	6, 12, and 24 h	Upregulation of AQP4 expression via p38 MAPK pathway.	[80]
**SFN**	THP-1 cells treated with Aβ1–42 (10 μM) for 8 h	5 μM	pretreated for 30 min	Decreased MerTK expression through inhibiting NF-κB nuclear translocation.	[81]
**SFN**	Primary microglia were prepared from 1-day-old C57 mice treated with Aβ (50 μM) for 24 h; primary cortical neurons from 1-day-old C57 mice treated with Aβ (50 μM) for 24 h; microglial cell line (BV-2) treated with Aβ (50 μM) for 24 h	1 μM	For 24 h	SFN inhibits NLRP3 inflammasome activation in Aβ-activated microglia and suppresses ROS accumulation.	[82]
**SFN**	BV-2 cells treated with fAβ25–35 (50 μmol/L) for 24 h	10 μmol/L	24 h	SFN reverses M1-type microglia polarization by downregulating the MAPK/NF-κB signaling pathway in Aβ25–35-activated BV-2 cells.	[83]
**SFN**	Primary cortical neuronal derived by ICT mouse	10 or 20 μM	3–6 h	Epigenetic modification of BDNF.	[84]
**SFN**	SH-SY5Y treated with MGO (0.5 mM)	2.5 μM	24 h	Decreased MAPK activation. Reduced oxidative stress and increased intracellular GSH levels.	[85]
**SFN**	Primary cortical neuron cultures treated with Aβ (10 μM) for 48 h	0.1 µM	48 h	Maintenance of neuronal dendritic integrity.	[86]
**SFN**	SH-SY5Y treated with Aβ_25−35_ (20 μM) for 24 h	2 mΜ	3 h	Increased expression of p75NTR and decreased expression of HDAC1 and HDAC3.	[87]
**SFN**	BV-2 cells treated with LPS (1 μg/mL) for 23 h	0.5–32 μM	24 h	Decreased levels of NO, TNF-α, and IL-6, as well as NF-κB activation.	[89]
**SFN**	THP-1 cells treated with Aβ (5, 10, 20 μM) for 24 h	1, 2, 5 µM	24 h	Inhibition of inflammasome.	[90]
**SFN**	SH-SY5Y treated with Aβ (10 µM) for 24 h	1, 2.5, 5 μM	24 h	Inhibition of apoptosis and Aβ-induced DNA damage.	[94]
**6-MSITC**	SH-SY5Y transfected with pCDNA3.1-^NRF2ΔETGE^-V5	9 μM	6–16 h	Increased protein and mRNA levels of ADAM17.	[104]
**MOR**	SH-SY5Y treated with Aβ_1–42_ (10 µM) for 24 h	0.5 µM	24 h	Modulated the expression of genes involved in autophagy and senescence.	[108]
**MOR**	hPDLSCs	0.5 µM	48 h	Downregulation of the genes involved in mitophagy.	[109]
**Memit**	H9-derived NSCs treated with a cocktail of LPS and TNF-α (50 ng/mL for 16 h)	10 µM	24 h	Reduction in ROS production. Reduction in apoptosis.	[110]
**H_2_S Hybrid Compounds**	BV-2 cells treated with LPS (5 μg/mL for 24 h); SH-SY5Y treated with H_2_O_2_ or t-BuOOH (50 Μm for 30 min)	1 and 5 μM	24 h	Anti-inflammatory action; reduction in ROS and NO levels.	[111]
**Compounds**	**In Vivo Model**	**Dose**	**Treatment Duration**	**Main Findings**	**Ref.**
**SFN**	3 × Tg-AD mouse and non-transgenic mouse	10 and 50 mg/kg	8 weeks	Increased level of CHIP and HSP70 and reduced the accumulation of Aβ and tau.	[78]
**SFN**	3 × Tg-AD mouse	10 mg/kg/day and 50 mg/kg/day	8 weeks	Epigenetic modification of BDNF.	[84]
**SFN**	PS1V97L transgenic mice	5 mg/kg	4 months	Improvements of cognitive deficits.	[86]
**FN**	APP/PS1 double-transgenic mice	25 mg/kg	5 months	Improved cognitive function.	[87]
**SFN**	C57BL/6 mice receiving aluminum-containing water (0.4 g/100 mL) and i.j. with 200 mg/kg d-galactose for 80 days	25 mg/kg	80 days	Ameliorated neurobehavioral deficits. Reduced Aβ deposits and peroxidation.	[88]
**SFN**	Adult male SD rats treated with STZ 5 μL injection (3 mg/kg) at day 1 and day 3	25 and 50 mg/kg	6 weeks	Inhibition of tau protein phosphorylation by modulating the PI3K/Akt/GSK-3β pathway.	[89]
**SFN**	Wistar rats injected with Aβ (5 μg/μL)	10–20 mg/kg	28 days	Reduction in oxidative stress and neuroinflammation.	[91]
**SFN**	Diabetic mice (db/db) and C57BLKS/J	1 mg/kg	28 days	Mitigated cognitive decline and hippocampal AD-like lesions. Increased Nrf2 level.	[92]
**SFN**	Sprague–Dawley rats with vascular cognitive impairment induced through permanent occlusion of bilateral common carotid arteries	10 mg/kg	After surgery twice a week for 42 days	Improved learning and memory function.	[93]
**SFN**	Kunming mice receiving aluminum-containing water (0.4 g/100 mL) and s.i. of 200 mg/kg d-galactose for 90 days	25 mg/kg	90 days	Reduced cholinergic neuron loss.	[95]
**SFN**	Sprague–Dawley rats with i.c.v injection of 10 μL AβOs (500 pmol for 3 days)	5 mg/kg	7 days	Reduction in neuroinflammation and oxidative stress. Effect on the seratoninergic system. Reduced memory impairment and depressive behavior.	[96]
**SFN**	6 adult male Swiss mice (SWR/J) treated with LPS (0.75 mg/kg) for 3 weeks	25 mg/kg	3 weeks	Reduction oin oxidative stress and accumulation of Aβ. Activation of the AMPK signaling pathway and reduction in caspase-3.	[99]
**SFN**	Sprague–Dawley (SD) rats treated with SCOP (1.5 mg/kg, i.p.) from 11th to 19th days	15 mg/kg	14 days	Improvements in behavioral and memory tests. Increased levels of BDNF and CREB, resulting in improvements of the hippocampal synaptic activity.	[100]
**SFN**	Zebra fish (Danio rerio) treated with SCOP (400 μM/L)	25 μM/L	1 h	Improvement in memory function.	[101]
**SFN**	Male wild-type (WT) C57BL/6J mice and Nrf2-KO mice receiving an LPS (0.5 mg/kg) i.p. injection 4 h prior to imaging of the cerebral microcirculation	5 mg/kg and 50 mg/kg	24 h prior to LPS/vehicle injection	Reduction in recruitment of leukocytes at the brain level by downregulating E-cadherin and VCAM.	[102]
**BRO**	*Caenorhabditis elegans*	0, 100, 500, 1000, 5000, 7500, and 10,000 μg/mL	24 h	Reduction in oxidative stress.	[103]
**6-MSITC**	App^NLGF^ mice; C57BL/6J strain WT	0.4 mg/mL	10 months	Improved cognitive function.	[105]
**6-MSITC**	C57Bl/6 mice receiving stereotaxic i.c.v. injection of Aβ_1–42_	5 mg/kg	10 days	Decreased apoptosis. Reduction in ROS. Restoration of GSH levels. Increased spatial learning memory.	[106]

Acetylcholinesterase (AChE); butyrylcholinesterase (BuChE); broccoli extract (BRO); histone deacetylases (HDACs); liquid chromatography/electrospray ionization mass spectrometry (LC/ESI-MS); methylglyoxal (MGO); 6-methylsulfinyl hexyl isothiocyanate (6-MSITC); SNP (sodium nitroprusside); streptozotocin (STZ) Romanesco broccoli (RB); purple broccoli (PB); amyloid-β (Abeta); amyloid-β oligomer (AbetaO); NSC; lipopolysaccharide (LPS); tumor necrosis factor alfa (TNFα); tert-butyl hydroperoxide (t-BuOOH); hydrogen peroxide (H_2_O_2_); 6-methylsulfinyl hexyl isothiocyanate (6-MSITC); broccoli byproducts extract (BRO); sulforaphane (SFN); moringin (MOR).

**Table 3:** Preclinical studies: results on the effects of glucosinolates or isothiocyanates treatments in PD models.

Compounds	Computational and Investigational Studies		Main Findings		Ref.
**SFN and ER**	LC-MS-MS		The two compounds have similar biotransformation processes.		[125]
**Compounds**	**In vitro model**	**Dose**	**Treatment Duration**	**Main findings**	**Ref.**
**Red cabbage extracts**	SH-SY5Y treated with α-Syn for 24 h	100 µg/mL	24 h	Reduction in neurotoxicity.	[114]
**SFN**	PC-12 cells treated with MPP (500 µmoL/L) for 24 h	2.5 µmol/L	24 h	Antioxidant activity by Nrf2 pathway.	[116]
**SFN**	PC-12 cells knockdown for Nrf2 transfected with siRNA (200 pmoL)	1–40 μM	9; 24 h	Activation of the Nrf2-ARE pathway.	[117]
**SFN**	SH-SY5Y treated with Rotenone (0.5 and 1 μM) for 24 h	10 μM	Pre-treatment for 2 h	Decreased oxidative stress, mTOR-dependent inhibition of neuronal apoptosis, and the restoration of normal autophagy.	[119]
**SFN**	SH-SY5Y, HEK293T, and primary neurons treated with MPP^+^ (1 mM)	1 μM	24 h	Reduction in C/EBPβ transcription; reduction of α-Syn expression and inflammation markers.	[120]
**SFN**	SH-SY5Y transfected with DJ1/LRRK2/scramble siRNA and treated with cypermethrin (15 μM) for 24 h	5 µM	30 min before the cypermethrinexposure	Increased levels of Nrf2, DJ-1, Prx3, and Trx2. Decreased apoptosis.	[121]
**SFN**	SH-SY5Y treated with MPP+ (1 mM) treated for 24 h	1 μM	24 h	Increased levels of Nrf2 and BDNF proteins. Decreased MeCP2 protein expression.	[124]
**SFN or ER**	SH-SY5Y treated with 6-OHDA (100 μM) for 24 h	5 µM	24 h	Modulated mTOR expression. Increased Nrf2 and GSH levels.	[126]
**GMG or MOR**	RAW 264.7 treated with LPS (10 μg/mL) for 24 h	GMG: 1 mg/mL; MOR obtained by GMG+MYR: 20 μL/mL	Pre-treatment for 2 h. After LPS for 15 min.	Reduction in pro-inflammatory cytokines including TNF-α and IL-1β. Reduction in TLR4 receptor activation.	[128]
**ITC-3**	BV-2 cells and DAergic CATH.a neuronal cells treated with LPS (0.2 μg/mL) for 24 h	1–20 µM	24 h	Reduction in pro-inflammatory cytokines. Increased Nrf2 levels and the antioxidant enzymes NQO1, HO-1, and GCL.	[129]
**ITC-57**	BV-2 cells treated with LPS (0.2 μg/mL) for 24 h andDAergic CATH.a neuronal cells exposed to BH4 or H_2_O_2_ (0.05–0.5 μM) for 24 h	0.5–3 μM	24 h	Reduction in inflammation.	[130]
**Compounds**	**In Vivo Model**	**Dose**	**Treatment Duration**	**Main Findings**	**Ref.**
**SFN**	C57BL/6N treated with MPTP (30 mg/kg) for 22 days	100 μM/kg	22 days	Reduction in dopaminergic neuron apoptosis and improvement of motor deficit.	[115]
**SFN**	Wistar rats treated with cypermethrin (1.5 mg/kg twice weekly) between days 5 and 19	10 mg/kg	twice weekly for 12 weeks, 1 h prior to cypermethrin treatment	Reduced oxidative stress and apoptotic neuronal cell death in the rat nigrostriatal tissue.	[121]
**GRA**	C57BL/6 mice treated with MPTP food pellet (10 mg/kg for 28 days)	0.1% GRA food pellet	28 days	Increased levels of Nrf2 and antioxidant enzymes.	[118]
**SFN**	C57BL/6 mice treated with rotenone (30 mg/kg) for 60 days	50 mg/kg	60 days	Reduction in oxidative stress and apoptosis.	[119]
**GRA**	Transgenic mice expressing A53T human α-Syn treated with intrathecal administrations of C/EBPβ-HDO (100 nM per 2 μL) on Day 1 and Day 5 and contemporary MPTP (30 mg/kg for 5 days)	0.1%	30 days	Reduced degeneration of dopaminergic neurons.	[120]
**SFN**	Male adult C57BL/6 mice treated with MPTP (30 mg/kg) for 5 days	10 mg/kg	10 days	Reduction in dopaminergic neurotoxicity and neuroinflammation. Increased BDNF levels	[124]
**SFN or ER**	C57BL/6 mice treated with 6-OHDA (4 μg/μL) for 4 weeks	30 μmol/kg	4 weeks.	Improvement of motor deficits. Decreased death of dopaminergic neurons. Reduction in DNA fragmentation.	[126]
**ER**	*C. elegans*	100 µM and 200 µM	4 h	Reduction in α-Syn aggregation and improvement in motor capacity.	[127]
**GMG or MOR**	C57Bl/6 mice treated with MPTP (20 mg/kg) for 14 days	MOR obtained by activation of GMG: 10 mg/kg of GMG +5 μL MYR/mouse (20 μL/mL injection.); GMG: 10 mg/kg	Pre-treatment daily for 1 week	Compared with GRA, MOR had greater effects by reducing dopaminergic neuroxicity; it also reduced the apoptotic process and improved motor coordination.	[128]
**ITC-3**	C57Bl/6 mice treated with MPTP (4 injections with 2 h intervals, 20 mg/kg for 7 days)	30 mg/kg	7 days	Reduction in motor deficit; activates the pathway of Nrf2, which determines both anti-inflammatory and antioxidant effects.	[129]
**ITC-57**	C57BL/6J mice treated with MPTP (4 injections with 2 h intervals, 20 mg/kg for 7 days)	30 mg/kg	7 days	Improvement in motor deficits. Inhibition of inflammatory processes and oxidative stress.	[130]
**BITC**	Male Wistar rats treated with ZnSO_4_ (20 mg/kg twice a week for 2–12 weeks)	10 mg/kg/day 1 h before to ZnSO_4_	12 weeks	Reduced Zn-induced neurobehavioral anomalies. Restored the level of dopamine and its metabolites.	[131]
**6-MSITC**	C57Bl/6 mice treated with 6-OHDA (4 μg/mL for 4 weeks)	5 mg/kg	4 weeks	Reduction in neuroinflammation and stress. Improvement of motor deficits.	[132]

6-methylsulfinyl hexyl isothiocyanate (6-MSITC); sulforaphane (SFN); moringin (MOR); glucomoringin (GMG); benzyl isothiocyanate (BITC); 6-Hydroxydopamine hydrobromide (6-OHDA); α-synuclein (α-Syn); 1-methyl-4-phenyl-1,2,3,6-tetrahydropyridine (MPTP); euricin (ER); 1-methyl-4-phenylpyridinium (MPP+); liquid chromatography–tandem mass spectrometry (LC-MS-MS); tetrahydrobiopterin (BH4); hydrogen peroxide (H_2_O_2_);. CCAAT/enhancer-binding proteins β (C/EBPβ); DNA/RNA heteroduplex oligonucleotide (HDO); myrosinase (MYR); glucoraphanin (GRA).

**Table 4:** Preclinical studies: results on the effects of glucosinolates or isothiocyanates treatments in MS models.

Compounds	In Vitro Model	Dose	Treatment Duration	Main Findings	Ref.
**SFN**	OLN-93 cells treated with 200 µM tert-butyl hydrogen peroxide for 4 h	5–10 μM	24 h	Increased the expression of HO-1, NQO-1, and p62. Increased cell viability and oligodendrocyte progenitor cell differentiation.	[137]
**Synthetic ITCs compounds**	The Jurkat T-lymphocyte cell line transfected with Human MIF	Stock solutions (1 M) of ITCs the work concentration was not specified	30 min	Inhibition activity of MIF.	[138]
**Compounds**	**In Vivo Model**	**Dose**	**Treatment Duration**	**Main Findings**	**Ref.**
**SFN**	C57BL/6 mice treated with MOG (200 ng) for 14 days	50 mg/kg	28 days	Inhibition of inflammatory and demyelination processes.	[140]
**Bioactive GRA**	Male adult C57BL/6 mice in EAE model	10 mg/kg GRA + 5 µL/mouse of MYR enzyme	Pre-treatment for 7 days Post drug-treatment for further 15 days	Reduction in caspase 3 levels. Increased claudin-1, -3, and -5 as well as ZO-1, and reduction in the inflammatory process.	[141]
**SFX-01**	Female SJL mice in EAE model	10, 50 or 300 mg/kg	Prophylactic experiment for 19 days Therapeutic experiment for 40 days	Reduced residual disability after EAE onset;reduced apoptosis.	[142]
**GMG-ITC**	C57BL/6 mice in EAE model	10 mg/kg GMG + 5 μL/mouse MYR	Pretreatment once a day for 7 days Therapeutic experiment for 21 days	Modulation of MAP kinase pathway; reduction in the inflammatory process; reduction of apoptotic process.	[143]
**GMG-ITC**	C57Bl/6 mice in EAE model	10 mg/kg GMG + 5 μL/mouse MYR	28 days	Modulation of Wnt-beta catenin pathway. Blocking of the release of inflammation mediators including IL-1β, IL-6, and COX-2. Increased Nrf2 levels.	[144]
**MOR-based cream**	C57Bl/6 mice in EAE model	2% (*w*/*w*)	21 days	Reduction in pro-inflammatory cytokines (TNF-α, IFN-γ) and increased IL-10 levels. Reduction in neuropathic pain by blocking ion channels.	[145]

Sulforaphane (SFN); moringin (MOR); glucomoringin (GMG); glucoraphanin (GRA); myrosinase (MYR); macrophage migration inhibitory factor (MIF); autoimmune encephalomyelitis (EAE); myelin oligodendrocyte glycoprotein (MOG).

**Table 5:** Preclinical studies: results on the effects of glucosinolates or isothiocyanates treatments in ASD models.

Compounds	In Vitro Model	Dose	Treatment Duration	Main Findings	Ref.
**SFN**	Neutrophils from peripheral blood of patients with ASD	5 µM	30 min	Recovered Trx1/TrxR1 redox system; reduced ROS production induced by methylmercury.	[151]
**SFN@EVs**	hPC12 cells	2.25 mM SFN-loaded 0.021 mM EVs	24 h	Increased Nrf2 gene expression. Decreased IL-6 gene expression.	[152]
**Compounds**	**Ex Vivo Model**	**Dose**	**Treatment Duration**	**Main Findings**	**Ref.**
**SFN**	Human PBMCs LPS-treated human PBMCs Human PBMCs	2 or 5 µM 5 µM 0.5 µM	6 h Pre-treatment 30 min Every 24 h for 3 days	Increased expression of cytoprotective enzymes (NQO1, HO-1, AKR1C1). Decreased levels of pro-inflammatory markers (IL-6, IL-1β, COX-2 e, TNF-α) after stimulation with LPS. Increased expression of cytoprotective enzymes (NQO1 and AKR1C1).	[153]
**SFN**	LPS-treated monocytes isolated from blood of ASD subjects	5 µM	12–16 h	Increased Nrf2 expression. Reduced oxidative damage (iNOS, Nitrotyrosine). Upregulated antioxidant enzymes (SOD1, GPx1, GR). Reduction in NF-κB activation. Decreased pro-inflammatory cytokines levels (IL-6, IL-1β).	[154]
**Compounds**	**In Vivo Model**	**Dose**	**Treatment Duration**	**Main Findings**	**Ref.**
**SFN@EVs**	Brain of chick embryos exposed to sodium valproate	2.25 mM SFN-loaded 0.021 mM EVs	10 days	Increased survival rate compared to sodium valproate-exposed groups. Restoration of normal gray and white matter structure. Improved bipolar neuron orientation.	[152]
**SFN**	Male BTBR T + Itpr3tf/J (BTBR) C57BL/6 (C57) mice (8–10 weeks old)	50 mg/kg/day ≈ 282 μmol/kg/day 50 mg/kg/day	7 days 7 days	Reduction in self-grooming/marble burying behavior, as well as increased social interaction. Reduction in Th17 immune responses (STAT3, RORC, IL-17 A, and IL-23R expression in CD4+T cells). Reduction in oxidative stress parameters in neutrophils/cerebellum (NF- κ B, iNOS, and lipid peroxides). Overexpression of SOD, GPx, and GR.	[155]
**SFN**	MIA-induced ASD-like rats	20 µg/kg	28 days	Improving of the sniffing times. Changes in the gut microbiota composition.	[156]
**GRA**	MIA-induced pregnant and lactating ddY famale mice	0.1% GF pellets	16 days	Improvement in the time spent in chamber in juvenile offspring after MIA. Improvement in the retention test (NORT) in the adult offspring after MIA. Reduction in parvalbumin immunoreactivity in the mPFC in the adult offspring after MIA.	[157]

SFN loaded to EVs (SFN@EVs); Maternal Immune Activation (MIA); medial Prefrontal Cortex (mPFC); Novel Object Recognition Test (NORT).

**Table 6:** Clinical studies: results on the effects of glucosinolates or isothiocyanates treatments on behavior and health in individuals with ASD.

Study Type	Partecipants	Treatment	Daily Dose	Duration	Main Findings	Ref.
**Single-Arm Clinical Trial**	17 Children (aged 4–7) with ASD (*n* = 6) or healthy controls (*n* = 11)	GRA group: Tablet containing broccoli seed and sprout extracts as a source of GRA and active MYR enzyme (Avmacol^®^) Healthy control group: Not treated	≥30 μmoL of GRA per table	12 weeks	Improvement in verbal or non-verbal communication (OARS-4), but no changes in social interaction and repetitive/ritualistic behaviors. No association between gut microbial diversity and treatment. Correlation of 35 gut microbiome abundance changes with ASD symptoms.	[156]
**Phase II, double-blind, randomized, placebo-controlled trial**	44 young men (aged 13–27) with moderate to severe ASD (*n* = 29 SFN vs. *n* = 15 placebo)	SFN group: Capsules of SFN-rich broccoli sprout extracts Control group: Placebo capsules containing microcrystalline cellulose	50–150 µmoL of SFN once daily ≈1.41 μmoL/kg/day SFN ≈0.25 mg/kg/day SFN	18 weeks	Improvement in social interaction, abnormal behavior, and verbal communication (ABC, SRS-2, CGI-I).	[158]
**Phase III, open-label trial**	15 young men (aged 5–22) with ASD	Tablet containing broccoli seed and sprout extracts as a source of GRA and active MYR enzyme (Avmacol^®^)	≈5.5 μmoL/kg/day GRA ~ 2.2 μmoL/kg/day SFN	12 weeks	Improvement in social responsiveness related to 77 urinary metabolites.	[160]
**Double-blind, randomized, placebo-controlled**	60 young men (aged 4–12) with ASD (*n* = 30 SFN vs. *n* = 30 placebo)	SFN group: Risperidone + capsules containing SFN Control group: Risperidone + placebo caspules	50–100 µmoL of SFN once daily ≈2.14 μmoL/kg/day SFN ≈0.38 mg/kg/day SFN	10 weeks	Improvements in irritability and hyperactivity, but none in lethargy/social interaction, stereotypy, inappropriate speech (ABC), and frequent adverse events.	[161]
**Phase II, double-blind, randomized, placebo-controlled trial**	45 children (aged 3–12) with moderate or greater severity of ASD (*n* = 22 GRA vs. *n* = 23 placebo)	GRA group: Tablet containing broccoli seed and sprout extracts as a source of GRA and active MYR enzyme (Avmacol^®^) Control group: Placebo tables containing microcrystalline cellulose and magnesium stearate.	≈5.5 μmoL/kg/day GRA ~ 2.2 μmoL/kg/day SFN	30 weeks	Improvement in aberrant behavior. GSSG increased, while free GSH/GSSG and total GSH/GSSG decreased. IL-6, Il-1β, and TNF-α decreased.	[162]
**Double-blind, randomized, placebo-controlled trial**	28 children (aged 3–7) with ASD (*n* = 15 SFN vs. *n* = 13 placebo)	SFN group: A SFN-rich broccoli sprout/red radish sprout powder mix (BroccoPhane^®^) Control group: Powder containing spinach puree	50 µmoL of SFN once daily ≈1.41 μmoL/kg/day SFN ≈0.25 mg/kg/day SFN	36 weeks	No statistically significant differences between the SFN and placebo groups in the behavioral and cognitive symptom rating scales (ABC, SRS-2, and ADOS-2).	[163]
**Double-blind, randomized, placebo-controlled multi-center trial**	108 Children (aged 3–15) with ASD ASD (*n* = 60 GRA vs. *n* = 48 placebo)	GRA group: Tablet containing broccoli seed and sprout extracts as a source of GRA and active MYR enzyme (Avmacol^®^) Control group: Placebo tablets.	≥30 μmoL of GRA per table 2–8 tables/day dose based on body weight	12 weeks	Significant improvements in autism features (CGI-I) and (OARS-4). No significant effects on behavior (SRS, ABC, and RBS-R). Improvements higher in participants older than 10 years of age and effects spanned range of intelligence.	[164]

Aberrant Behavior Checklist Scores (ABC), Autism Diagnostic Observation Schedule-2 (ADOS-2), OSU Autism Rating Scale-DSM-IV (OARS-4), Social Responsiveness Scale-2 (SRS-2), Clinical Global Impression Improvement Scale (CGI-I). Avmacol^®^ (Nutramax Laboratories, Inc., Edgewood, MD, USA); BroccoPhane^®^ (Bioriginal, Den Bommel, The Netherlands, Europe).

**Table 7:** Preclinical studies: results on the effects of glucosinolates or isothiocyanates treatments on schizophrenia models.

Compounds	In Vitro Model	Dose	Treatment Duration	Main Findings	**Ref.**
**D, L-SFN**	iMGLC	5 μM	24 h	Inhibition of NF-κB pathway activation (downregulated FOS and IL-1β). Nrf2 pathway activation (upregulated GCLM and HMOX1). Enhanced microglia phagocytosis.	[170]
**Compounds**	**In Vivo Model**	**Dose**	**Treatment Duration**	**Main Findings**	**Ref.**
**SFN** **GRA(Pellets containing Broccoli sprout extract powder)**	For SFN: Male ICR mice (6 weeks old) treated with phencyclidine For GRA: Mice (4 weeks old) treated with phencyclidine	30 mg/kg/day of SFN 0.1% ≈2.3 mmoL GRA per 1 kg-diet	***Prophylactic treatment with SFN*** 10 days (once daily on days 1–5 and 8–12) ***Therapeutic treatment*** *with SFN* 14 days (once daily on days 15–28) ***Prophylactic treatment*** with GRA 28 days	***Prophylactic treatment with SFN***: Prevention of phencyclidine-induced cognitive deficits. Prevention of phencyclidine-induced reduction in the spine density in the mPFC and hippocampal CA1. Prevention of phencyclidine-induced oxidative stress (8-oxo-dG-positive cells reduction). ***Therapeutic treatment*** *with SFN:* Improvement in phencyclidine-induced cognitive deficits. ***Prophylactic treatment*** with GRA: Prevention of phencyclidine-induced cognitive deficits. Reduction in parvalbumina-positive cells in mPFC and CA1. Prevention of phencyclidine-induced increase in 8-oxo-dG-positive cells.	[171]
**GRA** **(Pellets containing Broccoli sprout extract powder)**	Juvenile offspring mice (4 weeks old) derived from poly(I:C)-treated pregnant ddY mice	0.1% ≈2.3 mmoL GRA per 1 kg-diet	28 days	Improvement in cognitive deficits in adult offspring after MIA. Prevention of parvalbumin immunoreactivity reduction in the prelimbic regions of mPFC in adult offspring after MIA. Normalization of centrosome-related gene expression in prefrontal cortex and hippocampus of adult offspring after MIA. Prevention of SfI1 mRNA overexpression in prefrontal cortex and hippocampus of adult offspring after MIA.	[172]
**SFN**	Female C57BL/6 J mice (8 weeks old) treated with OLZ	90 mg/kg/day	35 days	Decreased fat accumulation and inflammation in liver (ALT and AST). Decreased triglyceride accumulation. Restored NEFA levels. Increased hepatic glycogen storage. Decreased adducts of 4-HNE in liver. Nrf2 pathway activation (increased NQO1 mRNA gene expression). Reduced lipid peroxidation (4-HNE).	[173]
**AITC**	Female BALB/c mice (6–8 weeks old) treated with OLZ	7.5 or 15 mg/kg/day	42 days	Reduced body weight, as well as food and water intake. Reduced adiposity and liver weight. Improved energy expenditure. Reduced fasting blood glucose. Improved glucose tolerance. Decreased insulin resistance. Reduced total cholesterol, LDL cholesterol, and triglycerides in the serum. Increased HDL cholesterol in the serum. Reduced pro-inflammatory markers (NF-κB; IL-6, IFN-γ, TNF-α). Increased expression of orexigenic neuropeptides (AgRP and NPY).	[174]
**SFN**	Male Wistar rats treated with OLZ	30 mg/kg/day	21 days	Reduced body weight, BMI, and food intake. Improved blood pressure and lipidic profile (increased HDL and reduced LDL, VLDL, triglycerides, and total cholesterol). Improvement in liver function parameters (AST, ALT, TBIL). Reduced oxidative stress (increased SOD and catalises; reduced MDA and total nitrite). Reduced pro-inflammatory cytokine levels (NF-κB; IL-6, TNF-α).	[175]

Allyl-ITC (AITC); human-induced pluripotent stem cell (hiPSC); hiPSC-derived microglia-like cells (iMGLC); olanzapine (OLZ); high-fat diet (HFD); polyriboinosinic–polyribocytidilic acid (poly(I:C)).

**Table 8:** Clinical studies: results on the effects of glucosinolates or isothiocyanates treatments on behavior and health in individuals with schizophrenia.

Study Type	Participants	Treatment	Daily Dose	Duration of Intervention	Main Findings	Ref.
**Open-label** **Single-arm clinical trial**	7 patients (aged 20–65) with schizophrenia	Tablets containing GRA	30 mg/day GRA ≈68.5 μmoL/day GRA	8 weeks	Improvement in visual recognition, attention, and short-term memory (OCLT Accuracy). No significant changes in other cognitive parameters (CogSate Battery), PANSS Total Score, and serum BDNF levels.	[176]
**Double-blind, randomized, placebo-controlled trial**	58 patients (aged 18–65) with schizophrenia or schizoaffective disorders (*n* = 29 GRA vs. *n* = 29 placebo)	GRA group: Tablet containing broccoli seed extract as a source of GRA and active MYR enzyme from broccoli sprouts (Avmacol^®^) Control group: Placebo capsules containing no active ingredients.	Dose per tablet: 16 mg or 37 μmoL of GRA 6 tablets/day: 222 μmoL/day of GRA ≈ 96 mg/day of GRA 6 tablets/day: appro. 100 μmoL/day of SFN ≈ appro. 17.7 mg/day	16 weeks	No statistically significant differences in PANSS total scores, and improvement in MCCB cognitive scores between GRA and placebo groups.	[177]
**Double-blind, randomized, placebo-controlled trial**	172 patients (aged 18–50) with schizophrenia (*n* = 58 GRA High Dose, *n* = 56 GRA Low Dose, *n* = 58 placebo)	GRA High-Dose group: Tablet containing broccoli seed extract as a source of GRA and active MYR enzyme from broccoli sprouts (Avmacol^®^) GRA Low-Dose group: Tablet containing broccoli seed extract as a source of GRA and active MYR enzyme from broccoli sprouts (Avmacol^®^) Control group: 6 placebo tablets	GRA High-Dose group: 6 tablets/day ≈219 μmoL/day of GRA ≈99 μmoL/day of SFN SFN Low-Dose group: 4 tablets/day + 2 placebo tablets ≈146 μmoL/day of GRA ≈66 μmoL/day of SFN Control group: 6 placebo tablets	22 weeks	Improvement in spatial working memory, reasoning–problem solving, and verbal learning (MCCB Domain Score). No statistically significant improvement in several cognitive domains (MCCB overall composite score). No effects on PANSS total scores.	[178]
**Single arm clinical trial**	45 patients (aged 18–50) with schizophrenia	Tablets containing SFN (Nutramax^®^)	90 mg/day of SFN ≈507.4 μmol/day of SFN	24 weeks	Improvement in the PANSS negative subscale and PANSS total score. No change in the PANSS positive subscale. Increased HsCRP levels. Increase in SOD activity.	[179]

High-sensitivity C-Reactive Protein (HsCRP); MATRICS Consensus Cognitive Battery (MCCB); One Card Learning Task (OCLT); Positive and Negative Syndrome Scale (PANSS). Nutramax ^®^ (Nutramax Laboratories Consumer Care, Lancaster, SC, USA).

**Table 9:** Preclinical studies: results on the effects of glucosinolates or isothiocyanates treatments on depression/anxiety models.

Compounds	In Vitro Model	Dose	Treatment Duration	Main Findings	Ref.
**(R, S)-SFN**	PC12 cells treated with NGF	0.01, 0.1, 1 μM	4 days	Increased number of PC12 cells with neurite growth in a dose-dependent manner. Enhanced neurite growth by Nrf2 pathway activation.	[192]
**Compounds**	**In Vivo Model**	**Dose**	**Treatment Duration**	**Main Findings**	**Ref.**
**DL-SFN**	Male ICR mice	1–10 mg/kg/day	14 days(once daily)	Antidepressant-like effects: Reduced immobility time (FST, TST). Increased sucrose preference (SPT). Anxiolytic-like effects: Increased time in the central zone (OPT). Decreased latency to feeding (NSF). Reduced serum corticosterone and adrenocorticotropic hormone levels in stressed mice. Decreased serum IL-6 and TNF-α levels in stressed mice.	[146]
**(R, S)-SFN**	Male adult C57BL/6 mice (8 weeks old) CD1 mice (14 weeks old)	10 mg/kg	30 min	Prevented decrease in social interaction in stressed mice. Attenuated decreased sucrose preference in stressed mice. Increased BDNF levels in CA3, DG, and PFC, and decreased BDNF in NAc in stressed mice. Restored p-TrkB/TrkB ratio in CA3, DG, and PFC, and reduced p-TrkB/TrkB ratio in NAc in stressed mice. Increased levels of Nrf2 and decreased Keap1 in CA3, DG, and PFC	[192]
**GRA** **(Pellet containing broccoli sprout extract powder)**	Male C57BL/6 mice (5 weeks old)	0.1% ≈ 2.2 mmol GRA per 1 kg-diet	21 days	Prevented decreased social interaction in stressed mice.Attenuated decreased sucrose preference in stressed mice.	
**SFN**	Male adult C57BL/6 mice (8 weeks old)	3.0, 10, 30 mg/kg	1 day	Reduced serum TNF-α levels and microglia activation in the brain after LPS administration. Increased serum IL-10 levels. Antidepressant-like effects: Decreased TST and FST. Prevented LPS-induced reduction in BDNF, PSD-95, and GluA1 levels in the PFC, hippocampus, and NAs. Restored LPS-induced reductions in dendritic spine density in the PFC and hippocampus. Prevented excessive increases in dendritic spine density in the NAc. Antidepressant-like effects: Decreased immobility time in TST and FST after LPS administration.	[193]
**GRA** **(Pellets containing broccoli sprout extract powder)**	Male adult C57BL/6 mice (8 weeks old)	0.1% ≈ 2.3 mmol GAR per 1 kg-diet	21 days	Prevented LPS-induced reduction in BDNF, PSD-95, and GluA1 levels in the PFC and hippocampus. Restored LPS-induced reductions in dendritic spine density in the PFC and hippocampus. Prevented LPS-induced excessive increase in BDNF levels and dendritic spine density in the NAc.	
**SFN**	Male C57BL/6J mice	10 mg/kg	15 days (from days 14 to 28 post-surgery)	Reduced pain responses in neuropathic pain models through the Nrf2/HO-1 signaling activation and microglial activation inhibition. Alleviated mechanical allodynia and thermal hyperalgesia by reducing oxidative stress and modulating MAPK pathways. Decreased microglial activation and neuroinflammation by the downregulation of CD11b/c and suppression of JNK, ERK1/2, and p-38 phosphorylation. Restored neuronal function and neuroplasticity by the upregulation of Nrf2, HO-1, and related antioxidant enzymes. Enhanced the antiallodynic effects of morphine by preventing downregulation of µ-opioid receptors.	[194]
**SFN**	Adult male Sprague–Dawley rats (12 weeks)	5 mg/kg	7 days	Restored normal locomotor activity in OFT. Increased sucrose preference. Improved spatial learning and memory in the MWM. Decreased immobility time and increased jumping in the FST. Protected the serotonergic system by restoring TPH2 levels in the DRN. Normalized SERT levels in the striatum, preventing serotonin dysregulation. Reduced lipid peroxidation by decreasing MDA levels. Restored antioxidant defense by increasing GSH levels. Reduced pro-inflammatory cytokines, IL-1β and TNF-α, levels.	[96]
**GRA**	Male Wistar rats (8 weeks old)	50 mg/kg	30 min	Antidepressant effects: Reduced immobility and increased swimming frequency in FST. Restored 5-HT and NA levels. Decreased pro-inflammatory cytokines (NF-κB levels). Reduced ROS levels.	[195]
**AITC** **PITC**	Famale C57BL/6 mice with depression and anxiety associated with chronic osteoarthritis pain (6–8 weeks)	AITC: 4.4 μmoL/kg PITC: 13.3 μmoL/kg	4 days for AITC 10 days for PITC	Antidepressant effects (significant reduction in immobility time). Increased antioxidant enzymes levels (HO-1, NQO1, GSTM1, GSTA1).	[196]
**AITC** **PITC**	Male C57BL/6 mice with depression associated with neuropathic pain (5–6 weeks)	AITC: 4.4 μmoL/kg PITC: 13.3 μmoL/kg	4 days for AITC 13 days for PITC	Antidepressant-like effects: Reduced immobility time in TST in both nerve-damaged and nerve-undamaged mice.	[197]
**SFN**	Male adult exposed to LPS for 24 h: C57BL/6 mice (8 weeks old) CD1 mice (14 weeks old)	10 mg/kg	1 h	Antidepressant-like effects: Reduced immobility time in TST and FS by activation of Nrf2 and BDNF expression. Increased sEPSC frequency in the hippocampus by upregulation of BDNF and downregulation of its repressors (HDAC2, mSin3A, MeCP2). Increased Nrf2 and BDNF levels in mPFC and the hippocampus.	[198]
	Nrf2 homozygous KO mice (Nrf2−/−)	10 mg/kg	24 h	No changes in BDNF, synaptic activity, or depression-like behaviors in Nrf2 KO mice.	
**SFN**	Male adult exposed to social defeat stress: C57BL/6 mice (8 weeks old) CD1 mice (14 weeks old) Thy1-YFP mice	10 mg/kg	10 days	Increased BDNF expression in the mPFC. Promoted neural plasticity by restoring dendritic spine density. Reduced pro-inflammatory cytokines (TNF-α, IL-1β, IL-6). Increased anti-inflammatory cytokines (IL-10, IL-4). Microglial modulation: Inhibited pro-inflammatory phenotype (iNOS +).Activated anti-inflammatory phenotype (Arginase1 +). Increased social interaction (SIT). Reduced immobility time in the FST. Increased sucrose preference in the SPT.	[199]
**SFN**	Male adult exposed to social defeat stress: C57BL/6 mice (8 weeks old) CD1 mice (14 weeks old) Thy1-YFP mice	10 mg/kg	10 days	Activated anti-inflammatory phenotype (Arginase1 +) by increasing TREM2. Antidepressant-like effects: Increased social avoidance time. Increased SPT. Improved synaptic plasticity by BDNF-TrkB pathway modulation. Increased anti-inflammatory cytokines gene expression (IL-10, IL-4). Nrf2 pathway activation.	[200]
**AERSS rich in sulforaphene (0.001 mg/g)** **SFN (0.029 mg/g), iberin (5.037 mg/g dry weight)**	Swiss Webster mice	3, 10, 30, 100 mg/kg 500 mg/kg	30 min	Anxiolytic effect: Increased time spent in open arms (Elevated Plus Maze test). Reduction in vertical stretching behavior but no change in ambulatory activity.	[201]
**(R, S)-SFN**	Male C57BL/J6 mice subjected to OB (~8 weeks old)	2.5, 5, 10 mg/kg	14 days	Improvement in depressive-like behavior: Reduced hyperactivity and increased time spent in the central zone in the OFT. Reduced latency and increased time and number of grooming instances in the ST. Reduced ambulation and distance traveled in the LA. Enhanced SOD activity in the serum. Increased total antioxidant capacity (TAC) levels in the frontal cortex and serum.	[202]

Aqueous Extract of Raphanus sativus L. Sprouts (AERSS); β-Amyloid Oligomerers (AβOs); Allyl-isothiocyanate (AITC); Dorsal Raphe Nucleus (DRN); Elevated Plus Maze (EPM); Forced Swimming Test (FST); Hypothalamic–Pituitary–Adrenal (HPA); Spontaneous Locomotor Activity Test (LA); Morris Water Maze (MWM); Nerve Growth Factor (NGF); Novelty-Suppressed Feeding Test (NSF); Olfactory Bulbectomy (OB); Open-Field Test (OFT); Phenyl Isothiocyanate (PITC); spontaneous Excitatory Postsynaptic Current (sEPSC); Social Interaction Test (SIT), Sucrose Preference Test (SPT); Splash Test (ST); Total Antioxidant Capacity (TAC); Tail Suspension Test (TST); Yellow Fluorescent Protein (YFP).

**Table 10:** Preclinical studies: results on the effects of glucosinolates or isothiocyanates treatments on epilepsy models.

Compounds	In Vitro Model	Dose	Treatment Duration	Main Findings	Ref.
**SFN**	Mixed cortical neurons and glial cells cultures from postnatal (P0–P1) Sprague–Dawley rat pups	5 μM	1 day	Decreased ROS during epileptiform activity. Increased GSH levels. Decreased seizure-like activity-induced neuronal death.	[215]
**Compounds**	**In Vivo Model**	**Dose**	**Treatment Duration**	**Main Findings**	**Ref.**
**SFN**	Adult male Wistar rats Nrf2−/− and Nrf2+/+ mice	5 mg/kg/day in corn oil	15 days	Suppression of amygdale kindling progression. Improvement in oxidative stress and cognitive impairment induced by epileptic seizure by Nrf2 pathway activation.	[211]
**SFN**	Adult male CD1 mice	5 mg/kg/day in corn oil	5 days	Increased seizure thresholds and reduced the incidence of status epilepticus. Enhanced antioxidant defenses and improved mitochondrial respiration and ATP production in the hippocampus.	[212]
**D,L-SFN**	Albino Swiss mice	10–300 mg/kg/day in corn oil	1 day	Decreased seizure thresholds, procolvulsant effects, and leukopenia in mice induced by high doses of SFN (≥200 mg/kg). SFN (100 mg/kg) enhanced carbamazepine efficacy. Toxic doses caused sedation (at 150–300 mg/kg), hypothermia (at 150–300 mg/kg), impairment of motor coordination (at 200–300 mg/kg), decrease in skeletal muscle strength (at 250–300 mg/kg), and death (at 200–300 mg/kg).	[213]
**SFN or NAC+SFN**	Adult male Sprague–Dawley rats	5 mg/kg/day of SFN NAC (10 mg/ kg/day) + SFN (5 mg/kg/day)	SFN for 14 days or NAC + SFN for 7 days followed by SFN alone for 7 days or NAC + SFN for 4 days	Delayed seizure onset and frequency, as well as prevention of disease progression. Normalized hippocampal GSH/GSSG ratios. Prevented mitochondrial dysfunction, reduced oxidative damage, and attenuated HMGB1 translocation. Reduced oxidative stress during epileptogenesis.	[214]
**SFN**	Male and female Sprague–Dawley rats	5 mg/kg/day	5 days	Increased expression of Nrf2, HO-1, and NQO1. Restored GSH/GSSG ratio in cortex in the hippocampus. Increased total antioxidant capacity in plasma and the hippocampus. Decreased neuronal death in CA1 and CA3 hippocampal subfields.	[215]
**SFN**	10-day-old male Wistar rats	5 mg/kg/day	2 day (on day 10 and day 11)	Nrf2 pathway activation. Improved brain metabolic adaptation after status epilepticus. Normalized glucose metabolism, restored cerebral blood flow, and enhanced vascular responsiveness to stimulation. Supported neuroprotection via improved metabolic resilience.	[216]
**SFN**	Immature 12-day-old male Wistar rats	5 mg/kg/day	2 days (48 h and 24 h before SE)	Reduced oxidative stress and mitochondrial dysfunction following status epilepticus in immature rats. Attenuated superoxide production, 3-NT and 4-HNE levels, and partially preserved mitochondrial complex I activity. Nrf2-mediated neuroprotection.	[217]
**SIN**	Adult male Wistar rats	10 and 20 mg/kg every other day	1 month	Delayed seizure onset and reduced kindling progression in PTZ-kindled rats. Improved memory performance and increased antioxidant enzyme levels (SOD, CAT). Reduced hippocampal expression of pro-inflammatory genes Il1b and Nlrp3.	[218]
**BITC**	C57BL/6J mice	10 mg/kg/day in corn oil	7 days	Improved cognitive and motor function in epileptic mice and reduced neuronal loss, particularly in the cortex. Enhanced the expression of Nrf2, HO-1, and NQO1, increased antioxidant enzyme activity (GSH-Px), and reduced MDA levels.	[219]

Benzyl isothiocyanate (BITC); status epilepticus (SE); sinigrin (SIN); sulforaphane (SFN).

## Data Availability

Not applicable.

## Abbreviations

The following abbreviations are used in this manuscript:

AD	Alzheimer’s disease
PD	Parkinson’s disease
MS	Multiple sclerosis
ASD	Autism spectrum disorder
GLS	Glucosinolate
ITCs	Isothiocyanates
Nrf2	Nuclear factor 2-related erythroid-derived factor
ARE	Antioxidant response element
NF-κB	Nuclear factor-κB
SFN	Sulforaphane
MYR	Myrosinase
GRA	Glucoraphanin
SIN	Sinigrin
AITC	Allyl isothiocyanate
GST	Gluconasturtiin
PEITC	Phenethyl isothiocyanate
GER	Glucoerucin
ER	Erucin
GTL	Glucotropaeolin
BITC	Benzyl isothiocyanate
GMG	Glucomoringin
MOR	Moringin
GSH	Glutathione
GST	Glutathione S-transferases
GTP	Gamma-glutamyl transpeptidase
CG	Cysteinylglycinase
NAT	N-acetyl transferase
NAC-ITC	N-acetylcysteine isothiocyanate
CNS	Central nervous system
ROS	Reactive oxygen species
LPS	Lipopolysaccharide
IL	Interleukin
iNOS	Inducible nitric oxide synthase
COX	Cyclooxygenase
NO	Nitric oxide
PGE2	Prostaglandin E2
MGO	Methylglyoxal
CPF	Chlorpyrifos
HO-1	Heme oxygenase-1
BDNF	Brain-derived neurotrophic factor
OKA	Okadaic Acid
TNF	Tumor necrosis factor
TBI	Traumatic brain injury
MHE	Minimal hepatic encephalopathy
GMG-ITC	Glucomoringin-isothiocyanate
MMP	Metalloproteinase
Aβ	Amyloid β
RB	Romanesco broccoli
PB	Purple broccoli
ESI-MS	Electrospray ionization mass spectrometry
BACE1	β-site amyloid precursor protein cleaving enzyme 1
NQO1	NAD(P)H quinone oxidoreductase
SNP	Sodium nitroprusside
HSP	Heat shock protein
AQP4	Aquaporin-4
HDACs	Histone deacetylases
CVI	Cognitive vascular impartment
SCOP	Scopolamine
BRO	Broccoli extract
6-MSITC	6-methylsulfinyl hexyl isothiocyanate
ADAM17	Metalloproteinase 17
AChE	Acetylcholinesterase
LB	Lewy bodies
α-syn	α-synuclein
MPTP	1-methyl-4-phenyl-1,2,3,6-tetrahydropyridine
C/EBPβ	CCAAT/enhancer-binding protein β
MeCP2	Methyl-binding protein CpG2
RTT	Rett syndrome
6-OHDA	6-hydroxydopamine
TH	Tyrosine hydroxylase
BST	Broccoli seed tea
BBB	Blood–brain barrier
MIF	Migration inhibitory factor
EAE	Experimental autoimmune encephalitis
Trx1/TrxR1	Thioredoxin 1/thioredoxin reductase 1
EVs	Extracellular vesicles
PBMCs	Peripheral blood mononuclear cells
MIA	Maternal immune activation
mPFC	Medial prefrontal cortex
ABC	Aberrant Behavior Checklist
ADOS-2	Autism Diagnostic Observation Schedule-2
SRS-2	Social Responsiveness Scale-2
OARS-4	OSU Autism Rating Scale-DSM-IV
iMGLC	hiPSC-derived microglia-like cells
4-HNE	4-hydroxynonenal
OCLT	One Card Learning Task
MCCB	MATRICS Consensus Cognitive Battery
HPA	Hypothalamic–pituitary–adrenal
SSRIs	Selective serotonin reuptake inhibitors
TST	Tail suspension test
FST	Forced swim test
SPT	Sucrose preference test
OFT	Open field test
ACTH	Adrenocorticotropic hormone
DG	Dentate gyrus
PFC	Prefrontal cortex
NAc	Nucleus accumbens
AβOs	β-amyloid oligomers
PITC	Phenyl isothiocyanate
CSDS	Chronic social defeat stress
SIT	Social interaction test
TAC	Total antioxidant capacity
PCI	Percutaneous coronary intervention
TLE	Temporal lobe epilepsy
SE	Status epilepticus
CABG	Coronary artery bypass grafting
HAM-D	Hamilton Rating Scale for Depression
PAHs	Polycyclic aromatic hydrocarbons
